# miRNA expression profiles of the perilesional skin of atopic dermatitis and psoriasis patients are highly similar

**DOI:** 10.1038/s41598-022-27235-2

**Published:** 2022-12-31

**Authors:** Gemma Carreras-Badosa, Julia Maslovskaja, Helen Vaher, Laura Pajusaar, Tarmo Annilo, Freddy Lättekivi, Matthias Hübenthal, Elke Rodriguez, Stephan Weidinger, Külli Kingo, Ana Rebane

**Affiliations:** 1 Institute of Biomedicine and Translational Medicine, University of Tartu, Ravila 14B, 50411 Tartu, Estonia; 2 Endocrinology, Girona Biomedical Research Institute, Girona, Spain; 3 Institute of Genomics, University of Tartu, Tartu, Estonia; 4 Department of Dermatology, University Medical Center Schleswig-Holstein, Kiel, Germany; 5 Department of Dermatology, University of Tartu, Tartu, Estonia; 6 Dermatology Clinic, Tartu University Hospital, Tartu, Estonia

**Keywords:** Inflammation, Skin diseases

## Abstract

Atopic dermatitis (AD) and *psoriasis vulgaris* (PV) are chronic inflammatory skin diseases with heterogeneous molecular backgrounds. MicroRNAs (miRNAs) contribute to either development or regulation of many immune system related diseases. Only few miRNA profiling studies are available for AD and no comparisons between AD and PV skin miRNA profiles have been performed recently. We conducted a miRNA profiling analysis of skin, as well as serum, from adult AD and PV patients and control individuals. 130 miRNAs were differentially expressed in AD skin, of which 77 were common differentially expressed in AD and PV. No differentially expressed miRNAs were detected in serum. Pathway analyses revealed differentially expressed miRNAs to potentially target immune-system related pathways, including TNF-α, IL-2/STAT4 and IL-6/JAK/STAT3. Additional genetic analysis of published AD GWAS dataset detected association of several target genes of differentially expressed miRNAs in skin. Moreover, miR-28-5p, miR-31-5p, miR-378a-3p and miR-203a were validated as upregulated in the skin of AD and PV patients. All validated miRNAs were reliable predictive markers for AD or PV. In conclusion, miRNA expression pattern in the skin of adult AD patients is highly similar to that of PV with multiple differentially expressed miRNAs potentially involved in the regulation of immune responses in AD and PV.

## Introduction

Atopic dermatitis (AD) and *psoriasis vulgaris* (PV) are two most common chronic systemic inflammatory skin diseases^1,2^. AD is characterised by pruritus and eczema, while PV is characterised by sharply demarcated erythematous plaques covered by micaceous silvery-white scale. In developed countries, AD affects up to 5%^1^ and PV up to 3% of adults^2^. Complex and multifactorial pathogenesis comprise these two diseases^3^^4^ that present also altered adaptive immune responses. In AD, more strong Th2 responses occur and are considered to play triggering roles^1,3^. Accordingly, monoclonal antibodies binding to the IL-4 receptor-α subunit, and several JAK1 inhibitors, inhibiting both IL-4 and IL-13 signalling, have been demonstrated to be efficient in a large subset of patients with AD^5,6^. As for PV, enhanced Th17 responses are characteristic^2,4^; and monoclonal antibodies targeting interleukin (IL)-23, IL-17, and IL-17 receptor (IL-17RA), as well as TNF inhibitors, have been shown excellent efficacy^7,8^. Concordantly, several transcriptomic studies using lesional skin samples from AD and PV patients have confirmed more strong Th2 component in AD skin and enhanced Th17 cell-responses in PV, with still a remarkable overlap in differentially expressed genes in these two diseases^9,10^.

microRNAs (miRNAs) are small non-coding RNA molecules able to post-transcriptionally modulate gene expression, and to contribute to the development or regulation of several diseases, including skin inflammation^11^. Only few studies have aimed to describe miRNA expression profiles in the skin of AD patients^12,13^, while multiple studies have been performed for PV^14–16^. Moreover, to our knowledge, only the earliest study from 2007 compared miRNA profiles of AD and PV skin, and indicated both similarities and differences between these two diseases^17^. Several miRNAs have been shown to modulate immune and/or cellular responses either in AD or PV as for example miR-31^18^ and miR-146a^19,20^.

In addition to tissues, miRNAs have also been found in body fluids, such as serum or plasma, where their identification is of remarkable value as minimally invasive circulating markers of diseases^21^. Nevertheless, only a few studies from others describe miRNA expression profiles either in plasma or serum of children with AD^22^, or circulating miRNAs in serum samples of AD adult patients^23^ andin PV^24^.

Hereafter, although several miRNAs have been demonstrated to function in the regulation of disease related processes involved in AD and PV, there is no recent miRNA profiling data of AD skin, no serum miRNA profiling of adult European AD patients has been published to date, and comparison of AD and PV miRNA profiles and genetic analyses are scarce.

Our aim was to analyse the miRNA profile of skin and serum samples in AD and PV, as well as in control individuals, in order to assess the differentially expressed miRNAs in AD and PV vs controls. Additionally, we also wanted to assess differentially expressed miRNAs in AD as compared to PV. Finally, we aimed to perform genetic association analysis for miRNAs differentially expressed in skin of AD patients.

For that aim, we conducted a miRNA profiling analysis of both skin and serum samples from adult AD and PV patients and control individuals using a miRNA array, as well as further validation of selected miRNAs by RT-qPCR. Moreover, we performed genetic association analysis for the observed differentially expressed miRNAs in the skin of AD patients using an available GWAS AD dataset.

## Results

### Skin differential miRNA expression profiles of AD and PV are highly similar

**Figure 1:**
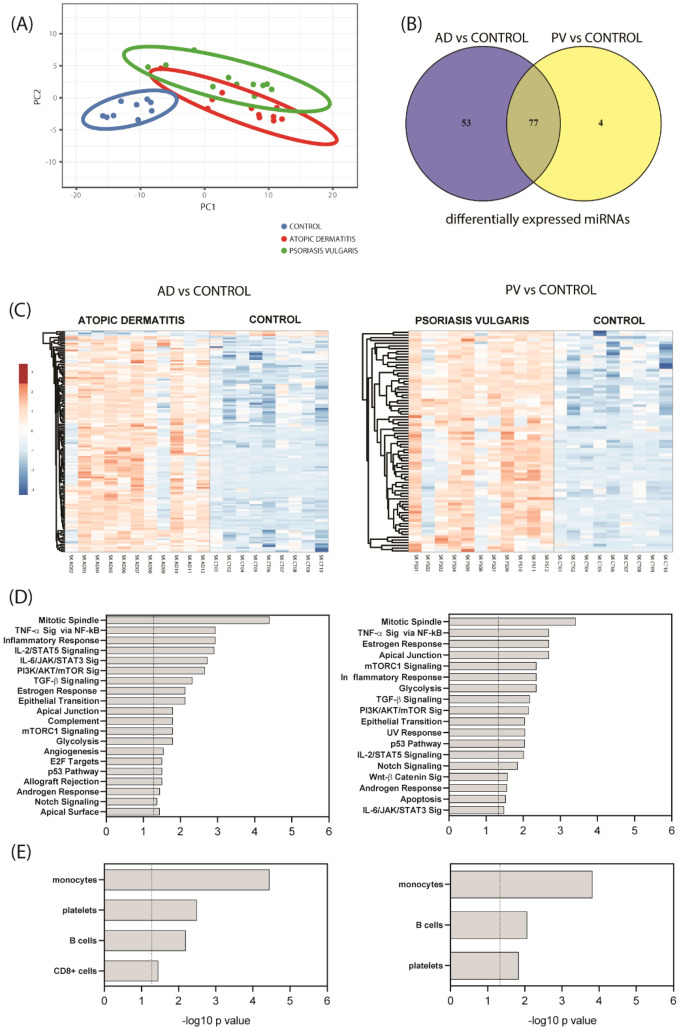
Differentially expressed miRNAs in skin tissue of AD and PV patients. (**A**) Principal component (PC) analysis of the miRNA profiling of the skin samples. Unit variance scaling is applied to rows; SVD with imputation is used to calculate principal components. X and Y axis show principal component 1 and principal component 2 that explain 64.2% and 6.3% of the total variance, respectively. Prediction ellipses are such that with probability 0.95, a new observation from the same group will fall inside the ellipse. N = 31 data points. (**B**) Venn diagram comparing the number of differentially expressed miRNAs from AD vs controls with PV vs controls analyses. (**C**) Hierarchical clustering analysis: heat map showing the differentially expressed miRNAs by studied groups plotted using ClustVis (correlation distance measure and average linkage function). Score values of principal component analysis (PCA) calculated from log2 values from the array miRNA expression signals were used. Clustered up- and down-regulated genes in AD and/or PV vs controls and AD vs PV from the profiling cohort. (**D**) Enrichment analysis of skin tissue samples for biological pathways performed with Enrichr. (**E**) Sites of expression of differentially expressed miRNAs-gene targets of skin tissue samples performed with Target Scan and Enrichr.

We conducted a miRNA profiling of skin samples from adult AD and PV patients and control individuals with comparable demographic characteristics (Table 1) using Affymetrix™ microarrays containing probes for 4604 human mature microRNAs, of which 2668 were detected to be expressed in skin. The principal component (PC) analysis of the miRNA expression of the samples showed an overlap on the AD and PV populations as compared to the controls (Fig. 1A). 130 miRNAs were identified as differentially expressed miRNAs in AD and 81 miRNAs as differentially expressed miRNAs in PV as compared to controls at differential FDR adjusted p-value ˂ 0.05, and log2 fold change > 2.0 or < − 2.0 (Fig. 1C). From all differentially expressed miRNAs, 127 were up-regulated and 3 down-regulated in AD and 81 up-regulated in PV as compared to controls (Supplementary Tables S1, S2).

**Table 1:** Characteristics of recruited AD and PV patients and age matched controls for the study of skin tissue samples.

	Profiling analysis				Validation analysis			
	All subjects	AD	PV	Controls	All subjects	AD	PV	Controls
	N = 31	N = 11	N = 11	N = 9	N = 33	N = 12	N = 12	N = 9
Age, years^A^	39.32 ± 13.92	36.27 ± 18.10	43.82 ± 12.53	37.56 ± 8.79	40.52 ± 14.44	37.50 ± 17.77	45.75 ± 13.69	37.56 ± 8.79
Sex, female, n (female %)^B^	14 (45.2%)	7 (63.6%)	3 (27.3%)	4 (44.4%)	14 (45.2%)	7 (58.3%)	3 (25.0%)	4 (44.4%)
Age of onset, years^A^			˂ 40				˂ 40	
**Severity of AD**								
moderate (IGA = 3), n (%)^B^		6 (54.5%)				7 (58.3%)		
severe (IGA = 4), n (%)^B^		5 (45.5%)				5 (41.7%)		
**Severity of PV, PASI score**^**A**^			10.23 ± 4.53				11.01 ± 5.09	
Affected joints, n (%)			7 (63.6%)				8 (66.7%)	
Affected nails, n (%)			5 (45.5%)				6 (50.0%)	
Affected head, n (%)			6 (54.5%)				7 (58.3%)	
Asthma, yes n (yes %)^B^	1 (3.2%)	1 (9.1%) ^#^	0 (0%)	0 (0%)	1 (3.0%)	1 (8.3%) ^#^	0 (0%)	0 (0%)
hsa-miR-31-5p relative signal, log2 or 2^-^^ΔCt^	4.83 ± 3.12	6.20 ± 0.92^	7.25 ± 0.63*^	0.19 ± 0.13	32.26 ± 28.10	21.19 ± 19.89	66.35 ± 38.46*^	1.58 ± 1.25
hsa-miR-378a-3p relative signal, log2 or 2^-^^Δ^^-Ct^	7.01 ± 0.69	7.29 ± 0.52^	7.40 ± 0.37^	6.18 ± 0.41	4.54 ± 2.92	4.97 ± 2.67^	6.63 ± 1.73^	1.16 ± 0.72
hsa-miR-28-5p relative signal, log2 or 2^-^^Δ^^-Ct^	2.88 ± 1.95	3.71 ± 1.79^	3.73 ± 1.47^	0.83 ± 0.71	3.53 ± 2.21	4.95 ± 2.10^	3.91 ± 1.59^	1.15 ± 0.68
hsa-miR-203a relative signal, log2 or 2^-^^Δ^^-Ct^	8.75 ± 0.67	8.96 ± 0.47^	9.21 ± 0.27^	7.92 ± 0.46	1.57 ± 0.90	1.27 ± 0.39*	2.27 ± 1.14^	1.03 ± 0.23

AD atopic dermatitis, PV psoriasis vulgaris, IGA investigator’s global assessment score for overall severity of AD signs, PASI psoriasis area severity index for overall severity of psoriasis signs.

^A^Data are shown as mean ± standard deviation (SD). ANOVA (post hoc test) *p˂0.05 comparing AD vs PV, ^ p˂0.05 compared to controls.

^B^Data are shown as absolute numbers and percentages in brackets. Chi Square test, ^#^p˂0.05.

Of note, previously identified deregulated miRNAs in AD and PV lesional skin have been identified here as well: including miR-31, miR-21, miR-203, miR-146a, miR-24, miR-27, miR-193 and miR-221^25,26^ (Supplementary Tables S1 and S2).

Moreover, among differentially expressed miRNAs, 77 were common in AD and PV as compared to healthy controls (Fig. 1B).

An additional analysis between AD and PV samples showed that no differentially expressed miRNAs could be found at differential FDR adjusted p-value ˂ 0.05, and log2 fold change > 2.0 or < − 2.0 (Supplementary Table S3). This demonstrates a striking similarity in miRNA expression profiles of skin biopsies from AD and PV patients.

We next performed the pathway analysis for the predicted targets of differentially expressed miRNAs. For AD vs controls, we detected among others very high enrichment for TNF-α via NF-κB, IL-2/STAT5 and IL-6/JAK/STAT3 signalling and; for PV vs controls we detected among others high enrichment for TNF-α via NF-κB and PI3K/AKT/mTOR signalling (Fig. 1D).

The enrichment analyses revealed that target genes of miRNA differentially expressed in AD and PV vs controls are expressed in monocytes and B cells, as well as in CD8 + cells in case of AD vs control (Fig. 1E).

### Genetic association analysis indicates no genetic association of differentially expressed miRNAs with AD but several polymorphisms are located in close vicinity to the target genes of skin AD differentially expressed miRNAs

Additionally, we screened the EAGLE eczema consortium GWAS dataset^27^ to assess whether polymorphisms known to be associated with AD fall within or close to the genes encoding differentially expressed miRNAs in order to identify miRNAs that may potentially have a genetic association with AD. This approach could help in identifying novel miRNAs that could be involved in AD pathophysiology.

We screened, for genetic associations (1) within and close to (± 30 Mb) the coding regions of the differentially expressed miRNAs and (2) within and close to (± 30 Mb) the coding regions of target genes of the differentially expressed miRNAs.

Being stringent to the significance threshold of the screening done between the previously mentioned available GWAS dataset related to AD and the list of genes encoding our differentially expressed miRNAs in skin of AD patients, results were as follows: , (1) we did not find a single signal in the coding region of the differentially expressed miRNAs, however, (2) we did find several polymorphisms within the coding regions or close vicinity of the target genes, including hsa-let-7f.-5p, -7 g-5p, -7i-5p target *IL13*, miRNA hsa-miR-130b-3p target *IRF1* and miRNA hsa-miR-28-5p target *STAT5B* (significance threshold p ˂ 0.05/10^–6^; output result shown in Supplementary Table S4 and Supplementary Fig. S1).

Out of the differentially expressed miRNAs, hsa-let-7f.-5p, -7 g-5p, -7i-5p, hsa-miR-130b-3p and hsa-miR-28-5p (Supplementary Table S1), and its respective target genes, IL13, IRF1 and STAT5B, were related to several polymorphisms involved in AD in the genetic association analysis (Supplementary Fig. S1). These results that observed polymorphisms within the coding regions or in close vicinity of the target genes of the differentially expressed miRNAs could suggest implications such as alterations on the binding capacity of these differentially expressed miRNAs to their target genes and/or affections in the promoter regions of their target genes, although further studies should be made in order to confirm such mechanisms.

### Skin expression of miR-31-5p, miR-28-5p, miR-378a-3p, miR-203a and miR-146a

Among the skin differentially expressed miRNAs, miR-31-5p, and miR-28-5p, miR-378a-3p, miR-203a and miR-146a were selected as candidates for validation. These miRNAs were selected because: (1) they were all differentially expressed miRNAs based on array data (Supplementary Tables S1 and S2) and (2) these miRNAs had been previously or in this study related to PV or AD More particularly, miR-31-5p was the top differentially expressed miRNA in both PV and AD samples in our study, and also extensively studied in PV previously^28^; miR-28-5p was identified as one of the differentially expressed genes in our array and in our genetic association analysis as miRNA targeting genes linked to AD; miR-378a-3p was selected because it is differentially expressed in AD and PV as well as because a recent study has described its relation to PV^29^; miR-203a was chosen as differentially expressed in AD and PV in our array dataset and because it has been also previously published relation to AD^30^ and PV^28^; and finally, miR-146a was selected as differentially expressed in the array data and it is known to inhibit inflammatory responses in both AD and PV^19,20,31^.

**Figure 2:**
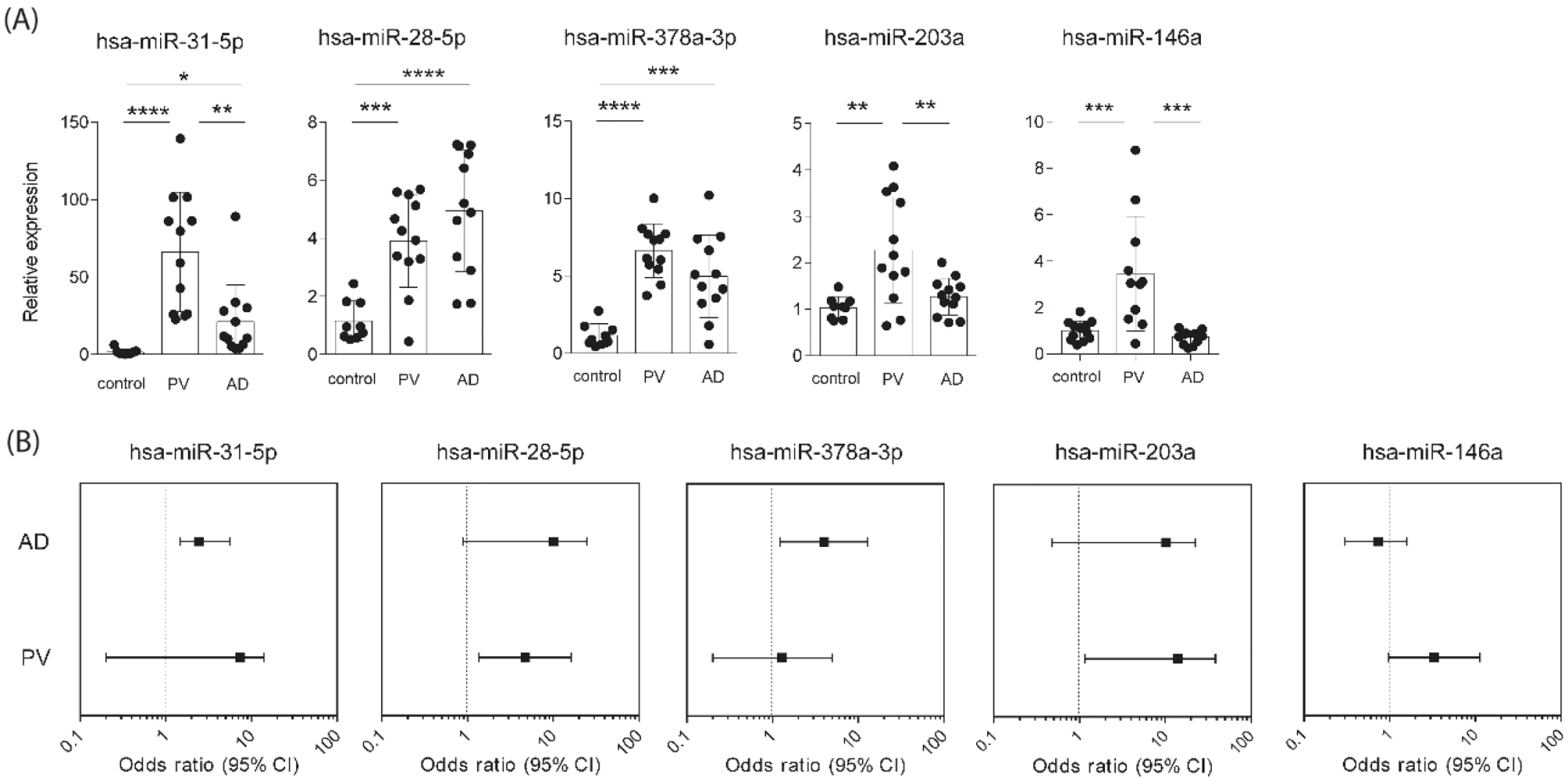
Skin miRNAs as predictive markers of AD and PV. (**A**) miR-31-5p, miR-28-5p, miR-378a-3p, miR-203a and miR-146a relative expression of control, PV and AD serum samples from the validation cohort assessed by RT-qPCR analysis. Data are represented as mean with SD and ANOVA (post hoc test) * p ˂ 0.05, ** p ˂ 0.01, *** p ˂ 0.001 and **** p ˂ 0.0001. (**B**) Forest plot showing Odds ratio (OR) and 95% confidence interval (CI) of miR-31-5p, miR-28-5p, miR-378a-3p, miR-203a and miR-146a for AD and PV assessed by logistic binary regression.

Figure 2A demonstrates that differentially expression of these selected miRNAs in AD and PV was confirmed in most cases by RT-qPCR. Moreover, the studied differentially expressed miRNAs were reliable predictive markers for AD or PV with a good or excellent discriminatory accuracy (good discriminatory accuracy with area under the curve (AUC) 0.899 ˃ AUC ˃ 0.700, excellent discriminatory accuracy with 1.000 ˃ AUC ˃ 0.900; Supplementary Fig. S2). Moreover, high levels of miR-31-5p (OR = 2.43; 95% confidence interval 1.04–5.64; p ˂ 0.05) and high levels of miR-378a-3p (OR = 3.97; 95% confidence interval 1.22–12.87; p ˂ 0.05) predicted the manifestation of AD (Fig. 2B). On the other hand, high levels of miR-28-5p (OR = 4.69; 95% confidence interval 1.35–16.33; p ˂ 0.05) and high levels of miR-203a (OR = 13.93; 95% confidence interval 1.15–38.45; p ˂ 0.05) were indicators of PV (Fig. 2B). Finally, levels of miR-146a did not predict the manifestation of AD or PV (Fig. 2B).

In conclusion, miRNA profiling results of skin biopsies from AD and PV patients revealed highly similar expression pattern between the two diseases as compared to controls.

### miRNA profiling of serum samples did not reveal any potentially differentially expressed miRNAs in AD and PV as compared to healthy controls

We next conducted a miRNA profiling of serum samples from adult AD and PV patients and control individuals with comparable demographic characteristics (Table 2) using the same Affymetrix microarrays as for the skin samples.

**Table 2:** Characteristics of recruited AD and PV patients and age matched controls for the study of serum samples.

	Profiling analysis				Validation analysis			
	All subjects	AD	PV	Controls	All subjects	AD	PV	Controls
	N = 49	N = 16	N = 16	N = 17	N = 70	N = 33	N = 19	N = 18
Age, years^A^	35.38 ± 9.75	34.62 ± 10.82	32.81 ± 9.62	38.52 ± 8.41	34.55 ± 10.99	32.78 ± 12.18	33.00 ± 9.46	39.44 ± 9.04
Sex female, n (female %)^B^	34 (69.4%)	11 (68.8%)	11 (68.8%)	12 (70.6%)	47 (67.1%)	21 (63.6%)	13 (68.4%)	13 (72.2%)
Age of onset, years^A^		9.68 ± 16.64*	20.18 ± 7.75			7.48 ± 12.42*	20.47 ± 7.79	
Disease duration, years^A^		23.66 ± 13.81*	11.06 ± 10.54			24.37 ± 14.03*	10.84 ± 9.81	
**Severity of AD**								
Moderate (IGA = 3), n (%)^B^		5 (31.3%)				10 (30.3%)		
Severe (IGA = 4), n (%)^B^		11 (68.8%)				23 (69.7%)		
Severity of PV, PASI score^A^			16.31 ± 6.14				15.66 ± 5.85	
Asthma, yes n (yes %)^B^	5 (10.2%)	5 (31.3%) ^#^	0 (0%)	0 (0%)	12 (17.1%)	11 (33.3%) ^#^	0 (0%)	1 (5.6%)
Allegic rhinitis, yes n (yes %)^B^	8 (16.3%)	8 (50%) ^#^	0 (0%)	0 (0%)	14 (20%)	14 (42.4%) ^#^	0 (0%)	0 (0%)
Hypertension, yes n (yes %)^B^	0 (0%)	0 (0%)	0 (0%)	0 (0%)	3 (4.3%)	2 (6.1%)	0 (0%)	1 (5.6%)
Anaemia, yes n (yes %)^B^	3 (6.1%)	1 (6.3%)	1 (6.3%)	1 (5.9%)	5 (7.1%)	3 (9.1%)	1 (5.3%)	1 (5.6%)
Impetigo, yes n (yes %)^B^	1 (2.0%)	0 (0%)	0 (0%)	1 (5.9%)	2 (2.9%)	1 (3%)	0 (0%)	1 (5.6%)
Acne, yes n (yes %)^B^	1 (2.0%)	0 (0%)	1 (6.3%)	0 (0%)	4 (5.7%)	2 (6.1%)	2 (10.5%)	0 (0%)
IgE, ng/ml^A^	364.48 ± 233.78	609.72 ± 559.02^	213.31 ± 117.58	149.49 ± 86.86	376.87 ± 246.97	526.37 ± 506.89*^	200.98 ± 182.92	149.49 ± 86.86
IL-12p40, pg/ml^A^	72.41 ± 34.14	64.30 ± 36.78	81.26 ± 33.70	71.72 ± 31.91	71.63 ± 32.90	68.91 ± 34.66	77.36 ± 32.19	70.56 ± 31,34
hsa-miR-122-5p relative signal, log2 or 2^-^^Δ^^-^^Ct^	1.17 ± 1.12	0.49 ± 0.35*	1.68 ± 1.09	1.32 ± 1.12	1.35 ± 1.38	0.97 ± 0.70*^	1.62 ± 1.28	1.78 ± 1.14

AD atopic dermatitis, PV psoriasis vulgaris, IGA investigator’s global assessment score for overall severity of AD signs, PASI psoriasis area severity index for overall severity of psoriasis signs.

^A^Data are shown as mean ± standard deviation (SD). ANOVA (post hoc test) *p˂0.05 comparing AD vs PV, ^ p˂0.05 comparing AD vs controls.

^B^Data are shown as absolute numbers and percentages in brackets. Chi Square test, ^#^p˂0.05.

The principal component (PC) analysis of the miRNA expression of the samples showed an overlap on the AD and PV populations as compared to the healthy controls (Supplementary Fig. S3A). We did not identify any differentially expressed miRNA in AD or PV as compared to controls, nor any differentially expressed miRNA in AD as compared to PV, for FDR adjusted p-value ˂ 0.05 and log2 fold change > 1.4 or < − 1.4 (Supplementary Tables S5, S6 and S7).

Despite previous studies have shown differentially regulated miRNAs in both plasma and whole blood from PV patients^32^, we did not find FDR adjusted p-value differences in serum samples between PV and healthy control samples of our cohort.

Nevertheless, unadjusted p-value ˂ 0.05 and log2 fold change > 1.4 or < − 1.4 showed potential differences: 9 miRNAs tended to be up-or-down-regulated in AD as compared to controls (Supplementary Table S5); 2 miRNAs tended to be down-regulated in PV as compared to controls (Supplementary Table S6); and finally 8 miRNAs tended to be up-or-down-regulated in AD patients as compared to PV patients (Supplementary Table S7).

To assess whether these potential deregulated miRNAs may be relevant to the disease, we performed bioinformatics analysis of their target genes. As a result, enrichment for PI3K/Akt/mTOR, IL-2/STAT5, TGF-β and TNF-α signalling, among other pathways was detected (Supplementary Fig. S3B). The enrichment analyses revealed those target genes to be expressed in blood vessels and skin among the top predicted tissues, and in B cells, NK cells, CD8 + cells, monocytes and CD4 + cells (Supplementary Fig. S3C).

Additionally, and as we had a serum validation cohort, we checked the potential deregulated miRNAs in serum by RT-qPCR and found that miR-122-5p was reduced in serum of adult AD patients as compared to PV and control subjects (Supplementary Fig. S4A) and confirmed to be a reliable predictive marker for AD outcome with a good discriminatory accuracy (Supplementary Fig. S4B). Moreover, low levels of circulating miR-122-5p predicted the manifestation of AD (OR = 1.79; 95% confidence interval 1.01–3.19; p ˂ 0.05; Supplementary Fig. S4C). Finally, AD patients, showing reduced miR-122-5p levels, also showed increased IgE levels in serum as compared to PV and control subjects while no differences were observed in the circulating levels of IL-12p40 among groups (Supplementary Fig. S4D).

## Discussion

### Skin differential miRNA expression profiles of AD and PV in the skin

We identified a highly similar differentially expressed miRNA profile in the skin of AD and PV patients, with 77 common differentially expressed miRNAs in AD and PV as compared to controls.

Both diseases, despite having clear different pathological and environmental triggers, are systemic inflammatory skin disorders that share some clinical characteristics (rashes or patches of red, raised, itchy skin) and are driven by T-cells^33^.

In AD, multiple phenotypes have been described, such as intrinsic AD, Asian AD, pediatric AD and European-American AD, however Th2 and Th22 cells are activated across all phenotypes^34^.

In PV, IL-12 and IL-23 drive T-cell axes Th1, Th17 and Th22 and activate transcription factors in keratinocytes to drive the immune responses^35^. The role of Th17, thought to be more related to PV, has been shown to be essential in concomitant AD and PV occurring at the same time in the same individuals^36^. Of note, the IL-23/Th17 axis is regulated by JAK/STAT3 pathway^37^, a pathway which also appeared in our enrichment analyses. Interestingly, this pathway is targeted in treatment of both PV^38^ and AD^39^.

Some speculations on the dashingly similar expression profile in the skin could be because of (1) similarities in skin cell responses and (2) due to the nature of our sampling, as all patient samples were collected at exacerbation stage from relatively severe patients prior to hospitalization. Interestingly, a recent study combining skin transcriptome and microbiome results reports that IL-1 family cytokines, including IL36G, which is considered PV specific gene^10^, to be upregulated in lesional skin of AD patients with more severe disease and high colonization levels of *Staphylococcus aureus* (*S. aureus*)^40^. In our study, we do not have information about *S. aureus* colonization rate, however, all recruited patients belonged to more sever disease groups with IGA 3 and 4 and we did not find any relationship between disease severity and the expression levels of miRNAs.

Genetic association analysis showed that the differentially expressed miRNAs were not genetically associated with AD. Thus we could speculate that the differential miRNA expression may be caused by the tissue responses during the disease course. However, we detected several polymorphisms to be significantly associated with the coding regions or close vicinity of the differentially expressed miRNA target genes, including hsa-let-7f.-5p, -7 g-5p, -7i-5p target gene IL13 known to be involved in processes such as recruitment of inflammatory cells, alteration of skin microbiome and impairment of epidermal barrier function^41^.

Among the selected miRNAs for validation, miR-31-5p, already reported to be upregulated in PV^42^, was validated in our study as upregulated in PV skin samples and to a lesser extent in AD. Similarly, miR-203a as upregulated in skin of PV patients. And the other two chosen miRNAs, miR-28-5p and miR-378a-3p were newly identified as upregulated in skin of both AD and PV patients.

Interestingly, miR-28-5p has been shown to target STAT5B, which has been related to the regulation of memory T cells^43^ and to the activation of mast cells in skin inflammation^44^. As STAT5B has shown to be required for an optimal IL-22 production under inflammatory conditions^45^, as well as it has been related to severity of eczema^46^, it may be implicated both in AD or PV. Furthermore, IL-34, related to Th1/Th17 cytokine production^47^, has been identified as miR-28-5p direct target.

miR-378a-3p was shown to be upregulated in PV^48^, by IL-17A in keratinocytes^49^, and has been shown to regulate keratinocyte responsiveness in PV^49^. We show here that miR-378a-3p is upregulated also in AD in similar extent as in the skin of PV patients. The role of miR-378a-3p in AD could be the regulation of IL-33 as miR-378a-3p has been described to exert a regulatory mechanism on IL-33 upon an inflammatory environment^50^, while IL-33 has been shown to be essential for inducing the immune responses to *S. aureus *in vivo and drive AD development^51^.

miR-203a has been described to promote cell proliferation through the activation of the PI3K/Akt signalling pathway^52^. Only Few publications have reported miR-203 to be involved in AD^22^, and our study suggests that miR-203a levels in the skin are significantly linked to PV^48^. In addition, a recent in vitro keratinocyte study revealed a regulation of miR-203a-3p in a PV-like manner by IL-24^15^.

Finally, miRNA 146a has been shown to act as anti-inflammatory miRNA in both PV and AD^19,20,31^. Here we detected the increased expression of miR-146a both in AD and PV using the array analysis, which was not confirmed by RT-qPCR in case of AD. As the fold change of miR-146a (PV versus control) was much higher in the array analysis as compared RT-qPCR, it is possible that miR-146a RT-qPCR probes are less sensitive.

Taken together, our comparative analysis of AD and PV skin samples shows that the expression of majority of miRNA are changed in both diseases, indicating that they may have a function in the regulation of skin responses of both AD and PV. Further studies are needed to delineate the functions of these miRNAs especially in association of AD which has been less studied.

### Serum miRNA expression in AD and PV

Although initially half of the study was focused on analysis of miRNA expression in the serum samples, we did not find significant differentially expressed miRNA profiles in AD or PV.

However, as previously other authors have described up-regulated serum miRNAs in AD in children’s cohorts^22^, we tested miR-122-5p and found it to be reduced in serum of AD patients. Changes in circulating miR-122-5p have been previously detected in many pathological conditions; it is known to induce proliferation and relieve lipopolysaccharide-induced inflammatory damage in keratinocytes^53^ and its downregulation has been related to pro-inflammatory cytokines and chemokines^54^. As previously high levels of miR-122-5p have been shown to promote Th1-associated M1 polarization^55^, the decrease in miR-122-5p in serum of AD patients could support the development of more strongTh2 responses in AD. In this line, our AD patients with reduced miR-122-5p in serum presented increased IgE levels as compared to PV and control subjects.

### Study strengths and limitations

Our study summarizes miRNA profiling results of both skin and serum samples of adult Caucasian AD and PV patients. This exploratory approach might provide useful information for future mechanistic studies to investigate on the role that these newly described miRNAs may have in AD or PV pathophysiology. As miRNA profiles in AD and PV from previous published studies have involved different age-groups, different methods and/or relatively small sample sizes; the replication and validation of miRNA profiles in other cohorts are of great interest and would be needed. Finally, data from miRNA profiling in serum has limitations: lower number of differentially expressed miRNA in the serum of AD and PV patients when compared to the skin, may be due larger individual variabilities of serum miRNA levels as well as a lower number of circulating miRNAs present in the serum samples as compared to tissue samples. The serum data was not significant after FDR adjusted p-value was used and other studies are needed with larger sample size and/or other techniques, such as small RNA sequencing. Another limitation is that we had available two separate cohorts, one for serum samples and another for the skin samples, so that data from and the skin and serum cannot be compared. In addition, we would like to point out that the current study is miRNA profiling study and all indications on miRNA functions are based on computer analysis and should be therefore further confirmed experimentally before conclusions about the roles of particular miRNAs can be made.

### Conclusion

To summarize, we report miRNA profiling analysis of skin and serum samples of adult AD and PV patients. Our results provide evidence of differentially expressed miRNAs in the skin, with a strikingly similarity of the two diseases according to miRNA expression changes. Moreover, we identified miR-378a-3p, and miR-28-5p and miR-203a as good candidates involved in the regulation of skin responses in AD and PV, respectively.

## Materials and methods

### Patient recruitment and samples

The study was approved by the Ethics Review Committee on Human Research of the University of Tartu and performed according to the Declaration of Helsinki Principles. All of the study participants were recruited among those seen Dermatology clinic in Tartu University Hospital (Estonia). Informed written consent was obtained from all of the patients.

The study population for skin sample donors consisted of thirty-three Caucasian patients, of which twelve subjects were diagnosed with AD and twelve with PV, and nine subjects were matched controls presenting non-inflammatory skin conditions (Table 1). The study population for serum samples consisted of seventy Caucasian patients, of which thirty-three subjects were diagnosed with AD, nineteen with PV, and eighteen subjects were matched controls presenting non-inflammatory skin conditions (Table 2). The profiling and validation samples were partially overlapping.

Clinical examination was performed according to the protocol followed by skin tissue punch extraction or venous blood sampling. Age, sex, age of onset, disease duration (years of ongoing disease), degree of severity and information on comorbidities (asthma, allergic rhinitis, hypertension, anaemia, impetigo and acne) were recorded. Subjects with comorbidities, such as diabetes, arthritis or neoplasms were excluded. Overall assessment of the skin lesions in terms of the Investigator’s Global Assessment (IGA) ranging from clear (= 0) to severe (= 4) and focusing on signs of erythema, induration/papulation, lichenification and oozing/crusting was used to define the level of severity for AD^56^. Psoriasis Area and Severity Index (PASI)^57^ was used to define the level of severity of PV and varied from 0 to 72.0 with a threshold of PASI score ≤ 10 defined as mild PV and PASI score ˃ 10 as moderate-to-severe PV^57^. All skin and serum samples were collected at exacerbation stage. None of the patients used neither topical nor systemic medications for at least two weeks prior to the time of skin or serum sample collection. None of the patients had been treated with any biologicals before recruitment into the study.

For skin, one punch biopsy skin sample (3–4 mm in diameter) was taken from the marginal zone of lesional skin on the upper limb (mainly around the shoulder area) from the patients with AD or PV, and from a non-sun-exposed site on the upper limb (mainly around the shoulder area) from the control subjects presenting non-inflammatory skin conditions. The serum samples were obtained by standard venepuncture. Separation of serum was performed by centrifugation at 1500*g* for 15 min at 4 °C after 60 min clotting process at room temperature. All skin and serum samples were kept frozen at − 80 °C until assay.

### ELISA

Serum IgE and IL-12p40 levels were measured as markers of allergy (Th2-related) and inflammation (Th17-related), respectively^23^. They were analysed by ELISA using Human IgE Platinum ELISA-BMS2097 (ThermoFisher, Waltham, MA, USA) and IL-12/IL-23 ELISA MAX™ Deluxe Human (BioLegend, San Diego, CA, USA). The detection limit of IgE was 0.5 ng/ml and the detection limit of IL-12p40 was 30 pg/ml. Both ELISA kits’ intra-assay coefficients of variability were less than 8%.

### RNA purification

Total RNA (including miRNAs) was isolated from a skin tissue punch (3–4 mm in diameter) or 150 μl of serum samples using Qiazol (Qiagen, Hilden, Germany) reagent and Total RNA Zol-out kit (A&A Biotechnology, Gdynia, Poland), respectively, according to the manufacturer’s instructions with minor modifications. To maintain small RNA fraction in case of Total RNA Zol-out kit, a 5:4 ratio of isopropanol was added to the samples before loading onto kit columns.

### Profiling of skin and circulating miRNAs

MiRNA profiling was performed by Affymetrix™ high-throughput microarray plates (miRNA 4.1 array plate, probes for 4604 human mature miRNAs) using Gene Titan™ multi-channel instrument. 300 ng or 150 ng of total extracted RNA was used per each skin or serum sample, respectively. Protocol included hybridization, staining and washing steps and was performed following manufacturer’s instructions. Both quality control (QC) and differential expression analysis were performed using the TAC software (Applied). QC was performed to exclude any possible technical biases that could influence the results, signal background subtraction was applied, and intra and inter-sample variation was normalized using default control probes and RMA-DABG method, with a detection p-value ˂ 0.05. QC also included PC analysis of samples (clustering for disease as expected) and correction by batch effects (such as sample plate and sex of the patients). In skin samples 2668 miRNAs (57.9%) were detected and in serum samples 707 miRNAs (15.3%) were detected. Processed data was fitted to a linear model. Differential miRNA expression analysis was performed using the eBayes moderated t-statistic and individual miRNAs p-values were derived using Limma package for R studio. Genes were considered to be differentially expressed for false discovery rate (FDR) adjusted p-value ˂ 0.05 and log2 fold change > 2.0 or < − 2.0 in skin samples and log2 fold change > 1.4 or < − 1.4 in serum samples due to the individual variabilities of serum samples.Hierarchical clustering analysis was performed and heat map was plotted using ClustVis web tool^58^.

Array data is available under GEO accession numbers GSE175438 for skin samples and GEO accession number GSE162926 for serum samples.

### RT-qPCR

MiRNA expression was validated by real-time PCR (RT-qPCR) on ViiA™ 7 Real-Time PCR system (Life Technologies, USA) and Taqman miRNA qPCR system. 10 ng of total RNA was reverse transcribed using TaqMan MiRNA Reverse Transcription Kit (Applied Biosystems, Foster City, USA) and miRNA specific primers. Real time PCR was performed in duplicates using 5 × HOT FIREPol® Probe qPCR Mix Plus (ROX) (Solis BioDyne, Tartu, Estonia) and the following Taqman miRNA assays: hsa-miR-31-5p (ID-002279), hsa-miR-28-5p (ID-000411), hsa-miR-378a-3p (ID-002243), hsa-miR-203a-3p (ID-000507), has-miR-146a-5p (ID-000468), hsa-miR-122-5p (ID-002245), hsa-miR-638 (ID-001582), SNORD48 (ID-001006) and hsa-let-7a (ID-000377) (Applied Biosystems, Foster City, USA). SNORD48 and let-7a were used as housekeeping genes for tissue samples. Hsa-miR-638 was highly and stable expressed in serum and used as reference for serum samples. Relative miRNA levels were calculated according to the 2^-ΔΔCt^ method, and the average expression of healthy controls was set to one.

### Pathway and genetic association analyses

Differentially expressed miRNA gene targets were screened using human conserved microRNA in Target Scan^59^. Enrichment analyses for predicting biological pathways and predicted tissue expression was performed using Enrichr tools^60^. To predict biological pathways, we used the Molecular Signatures Database (MSigDB)^61^ which is one of the most widely used and comprehensive databases of gene sets for performing gene set enrichment analysis. To predict tissues and cells influenced by differentially expressed miRNAs according to the target genes protein expression, we used the Genotype-Tissue Expression (GTEx) project^62^ database containing data on gene expression on every potential human tissue but no data on potential cells, and Human Proteome Map^63^ for several human tissues and cells, which contains specifically protein expression data on immune cells but lacks information on “skin” or “epithelium” tissues. Robustness of the enrichment analyses was assessed using random subsets of differential miRNAs and their target genes and observing that the predicted biological pathways and predicted tissue expressions observed in this study were specific of our differential set of miRNAs (data not shown).

Genetic association analysis overlapping the coding regions of candidate differentially expressed miRNAs from our study (analysis of AD vs controls) as well as their target genes (based on miRTarBase-8.0) with the previously available genetic data from EAGLE eczema consortium^27^ was performed with using a conservative significance threshold of p-value ˂ 0.05/10^6^ as previously described^64^. EAGLE eczema consortium is a published multi-ethnic GWAS dataset of 21,000 cases and 9500 controls that identified loci associated with AD^27^.

### Statistical analysis

Patient characteristics, clinical and analytic parameters, are expressed as mean ± standard deviation (SD) or percentage (calculated from absolute numbers). Variables were compared between groups by analysis of variance (ANOVA) with Bonferroni post hoc tests for quantitative variables or Chi-square test for qualitative categorical variables at confidence interval (CI) of 95%. To assess the diagnostic potential, the area under the curve (AUC) was calculated using receiver operating characteristic (ROC) curve and Odds Ratio (OR) values with confident interval (CI) 95% were calculated usinglogistic binary regression. In all cases, significance level was set to p-value ˂ 0.05. Data analyses were performed with the SPSS (v.21) statistical software and GraphPad (v.6). All figures were assembled using Adobe Illustrator software.

## Supplementary Information

Supplementary Information.

## Data Availability

The array datasets generated for this study will be publicly found in the GEO repository under GEO accession numbers: GSE175438 for skin samples and GEO accession number GSE162926 for serum samples. When needed, please contact the corresponding author for additional data.
