# Supplementary material for: miRNA expression profiles of the perilesional skin of atopic dermatitis and psoriasis patients are highly similar
Source: Sci Rep. 2022 Dec 31;12:22645. doi: 10.1038/s41598-022-27235-2 (PMC9805436; doi:10.1038/s41598-022-27235-2)
Supplement: Supplementary file 1 — Supplementary Information. [file 41598_2022_27235_MOESM1_ESM.pdf]

miRNA expression profiles of the perilesional skin of atopic dermatitis and psoriasis patients are highly similar

Gemma Carreras-Badosa[1,2], Julia Maslovskaja[1], Helen Vaher[1], Laura Pajusaar[1], Tarmo Annilo[3], Freddy Lättekivi[1], Matthias Hübenthal[4], Elke Rodriguez[4], Stephan Weidinger[4], Külli Kingo[5], Ana Rebane[1*]

[1]Institute of Biomedicine and Translational Medicine, University of Tartu, Tartu, Estonia

[2]Endocrinology, Girona Biomedical Research Institute, Girona, Spain;

[3]Institute of Genomics, University of Tartu, Tartu, Estonia

[4]Department of Dermatology, University Medical Center Schleswig-Holstein, Kiel, Germany

[5]Department of Dermatology, University of Tartu, Tartu, Estonia & Dermatology Clinic, Tartu University Hospital, Tartu, Estonia

*** Correspondence:**

Ana Rebane, PhD

Institute of Biomedicine and Translational Medicine

University of Tartu

Ravila 14B

50411 Tartu, Estonia

Tel: +372 7 374 419

Email: ana.rebane@ut.ee

Keywords: atopic dermatitis, biomarkers, inflammatory skin disease, microarray, microRNAs

1. **Supplementary Figure legends**

Supplementary Figure S1. Genetic association analysis of the DE miRNAs in skin tissue of AD patients. Regional plots showing the genetic associations from the EAGLE eczema consortium GWAS dataset and the coding regions of target genes of the AD DE miRNAs from our analysis. Polymorphisms found in the coding regions/closes vicinity of (A) IL13 (target gene for hsa-let-7f-5p, -7g-5p, -7i-5p), (B) IRF1 (target gene for hsa-miR-130b-3p) and (C) STAT5B (target gene for hsa-miR-28-5p) meeting the stringent threshold p-value $< 0.05/10^6$ are shown.

Supplementary Figure S2. Skin miRNAs as predictive markers of AD and PV. Diagnostic accuracy (ROC analyses) of miR-31-5p, miR-28-5p, miR-378a-3p, miR-203a and miR-146a for AD and PV (marked in bold an AUC from 0.9 to 1.0 considered acceptable for excellent discriminatory accuracy). For AD: 12 cases vs 9 controls, for PV: 12 cases vs 9 controls.

Supplementary Figure S3. Differentially expressed (DE) miRNAs in serum samples of AD and PV patients. (A) Principal component (PC) analysis of the miRNA profiling of the serum samples. Unit variance scaling is applied to rows; SVD with imputation is used to calculate principal components. X and Y axis show principal component 1 and principal component 2 that explain 32.5% and 17.3% of the total variance, respectively. Prediction ellipses are such that with probability 0.95, a new observation from the same group will fall inside the ellipse. N = 49 data points. (B) Enrichment analysis of serum samples for biological pathways performed with Enrichr. (C) Sites of expression of DE miRNAs-gene targets of serum samples performed with Target Scan and Enrichr.

Supplementary Figure S4. Circulating miR-122-5p is a predictive marker of AD. (A) miR-122-5p relative expression of control, PV and AD serum samples of the validation cohort assessed by RT-qPCR analysis. (B) Diagnostic accuracy (ROC analyses) of miR-122-5p for AD and PV (marked in bold an AUC from 0.7 to 0.8 considered acceptable for good discriminatory accuracy). For AD: 33 cases vs 18 controls; for PV: 19 cases vs 18 controls. (C) Forest plot showing Odds ratio (OR) and 95% confidence interval (CI) of miR-122-5p for AD and PV assessed by logistic binary regression. (D) IgE and IL-12p40 serum levels assessed by ELISA. Data are represented as mean with SEM and ANOVA (*post hoc* test) * $p < 0.05$.

2. **Supplementary table legends**

Supplementary Table S1. Differentially expressed skin tissue miRNAs in skin of AD vs control patients. Genes were considered to be differentially expressed (in bold) for FDR adjusted p-value < 0.05 and log2 fold change >2.0 or <-2.0

Supplementary Table S2. Differentially expressed skin tissue miRNAs in skin of PV vs control patients. Genes were considered to be differentially expressed (in bold) for p-value < 0.05, FDR adjusted p-value < 0.05 and log2 fold change >2.0 or <-2.0

Supplementary Table S3. Differentially expressed skin tissue miRNAs in skin of AD vs PV patients. No genes were considered to be differentially expressed (p-value < 0.05, FDR adjusted p-value < 0.05 and log2 fold change >2.0 or <-2.0)

Supplementary Table S4. Genetic association analysis of the DE skin tissue miRNAs in skin of AD, PV and control patients. Output results of the analyses between EAGLE eczema GWAS data set and genes encoding our studied DE miRNAs meeting the stringent threshold p-value $< 0.05/10^6$ are shown.

Supplementary Table S5. Differentially expressed serum miRNAs in serum of AD vs control patients. No genes were considered to be differentially expressed (FDR adjusted p-value < 0.05 and log2 fold change >1.4 or <-1.4)

Supplementary Table S6. Differentially expressed serum miRNAs in serum of PV vs control patients. No genes were considered to be differentially expressed (FDR adjusted p-value < 0.05 and log2 fold change >1.4 or <-1.4)

Supplementary Table S7. Differentially expressed serum miRNAs in serum of AD vs PV patients. No genes were considered to be differentially expressed (FDR adjusted p-value < 0.05 and log2 fold change >1.4 or <-1.4)

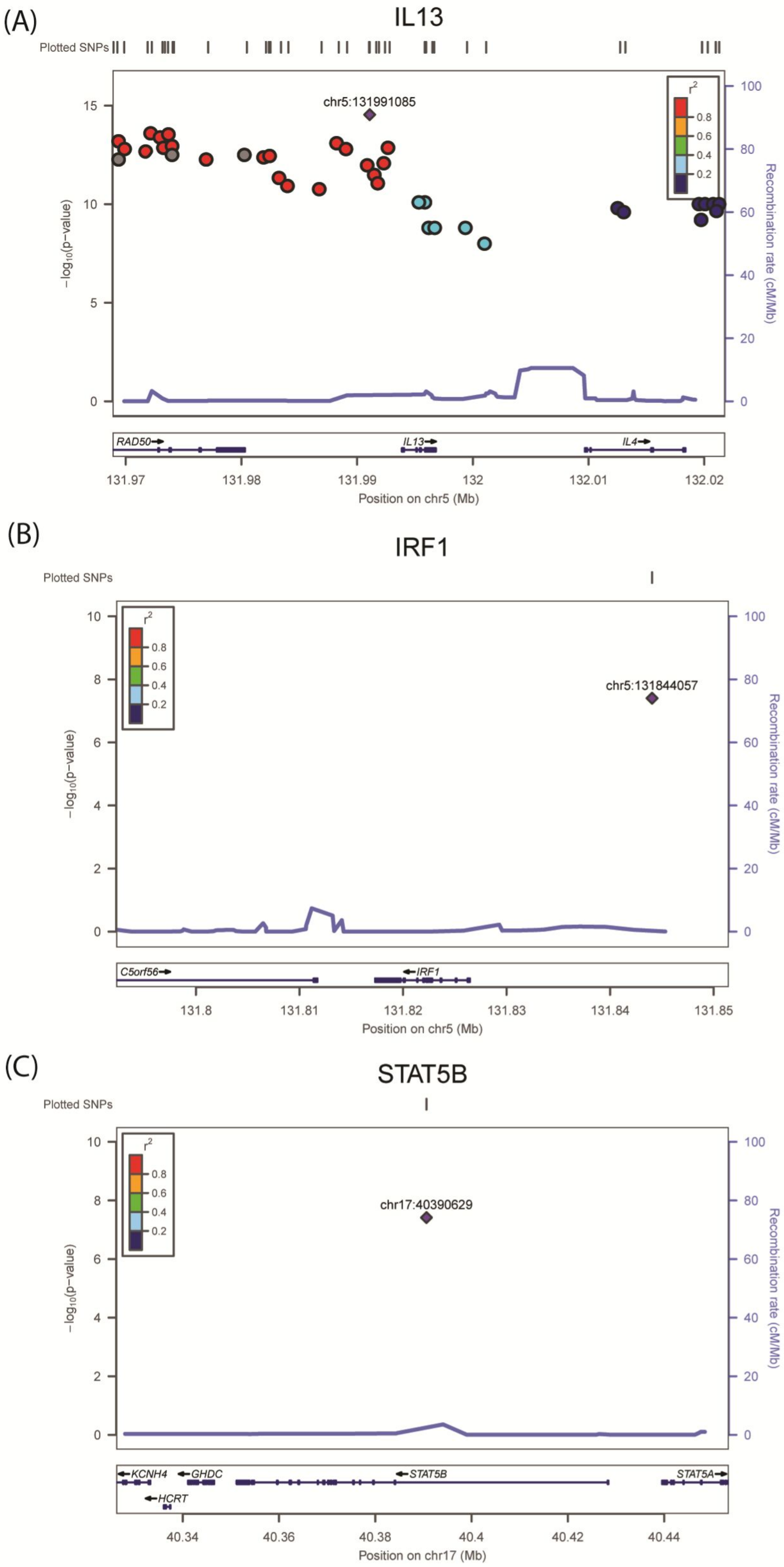

Supplementary Figure S1

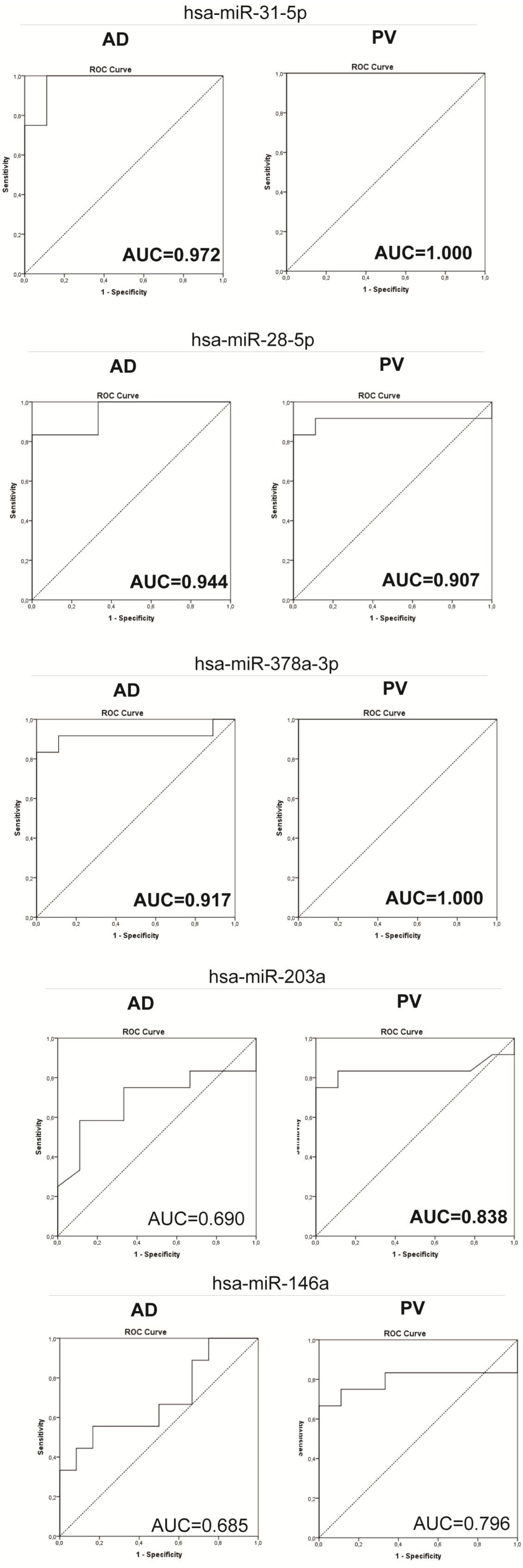

Supplementary Figure S2

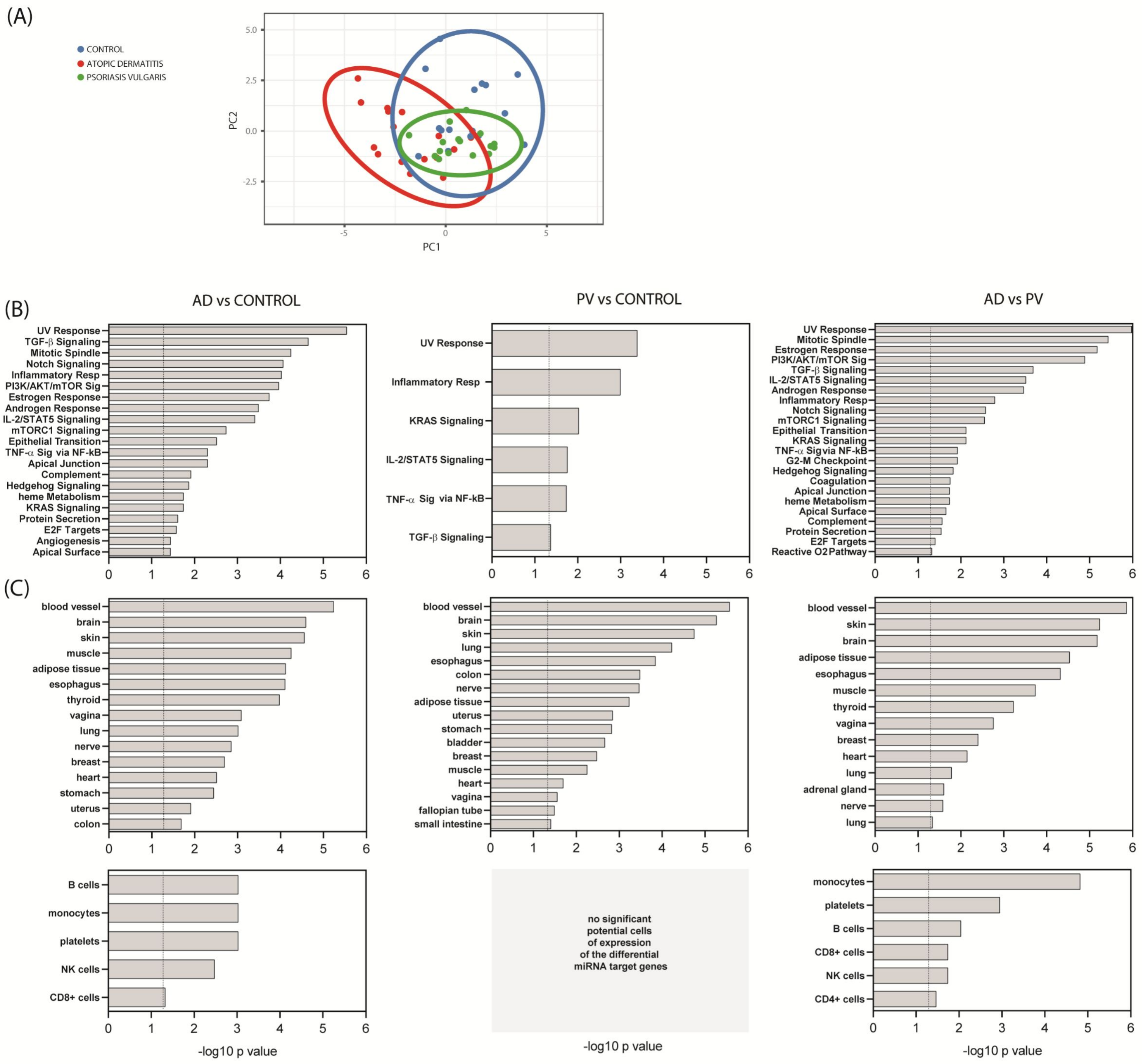

Supplementary Figure S3

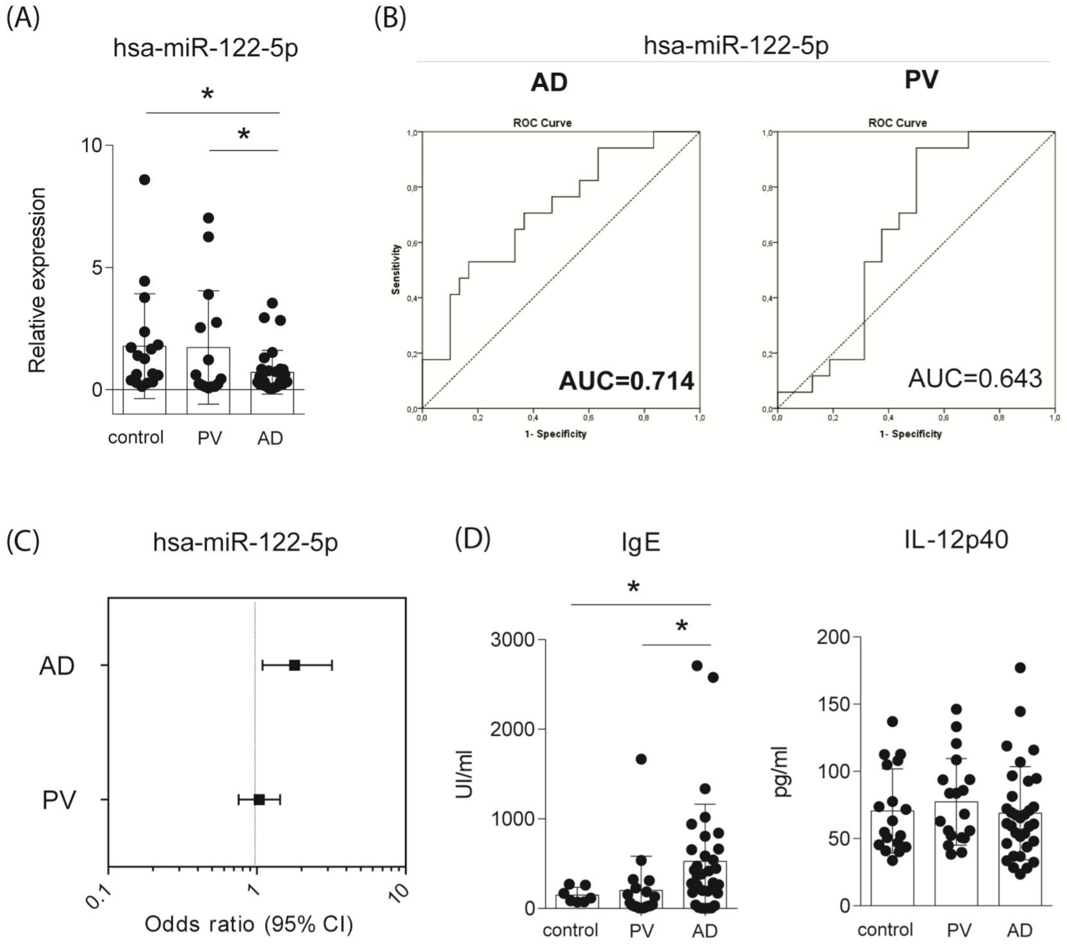

Supplementary Figure S4

Table S1

Transcript ID	array ID	Accession	Chromosome	Strand	AD (log2)	controls (log2)	AD vs controls		
							Fold Change	P-value	FDR adj p-value
hsa-miR-31-5p	20500164	MIMAT0000089	chr9	-	6,2	0,11	**68,05**	**5,10E-19**	**3,38E-15**
hsa-miR-6126	20524036	MIMAT0024599	chr16	-	4,96	0,07	**29,58**	**1,58E-06**	**0,0003**
hsa-miR-2115-5p	20511558	MIMAT0011158	chr3	-	4,76	0,3	**21,99**	**5,69E-12**	**1,89E-08**
hsa-miR-146a-5p	20500778	MIMAT0000449	chr5	+	5,18	0,73	**21,88**	**1,99E-07**	**5,72E-05**
hsa-miR-3175	20515603	MIMAT0015052	chr15	+	4,67	0,36	**19,79**	**2,54E-06**	**0,0004**
hsa-miR-3651	20517902	MIMAT0018071	chr9	-	4,8	0,53	**19,32**	**1,20E-07**	**4,69E-05**
hsa-miR-139-5p	20500432	MIMAT0000250	chr11	-	4,84	0,64	**18,33**	**0,0018**	**0,0343**
hsa-miR-25-3p	20500151	MIMAT0000081	chr7	-	5,28	1,1	**18,14**	**1,81E-06**	**0,0003**
hsa-miR-7977	20529568	MIMAT0031180	chr3	+	4,81	0,64	**18,02**	**2,05E-05**	**0,0017**
hsa-miR-92b-3p	20504274	MIMAT0003218	chr1	+	4,72	0,59	**17,44**	**4,28E-05**	**0,003**
hsa-miR-5100	20521785	MIMAT0022259	chr10	+	6,08	2,05	**16,28**	**1,31E-07**	**4,84E-05**
hsa-miR-532-3p	20503908	MIMAT0004780	chrX	+	4,55	0,55	**16,05**	**2,82E-06**	**0,0004**
hsa-miR-5189-3p	20520569	MIMAT0027088	chr16	+	4,12	0,14	**15,72**	**1,91E-05**	**0,0016**
hsa-miR-200b-3p	20500556	MIMAT0000318	chr1	+	4,7	0,74	**15,49**	**4,21E-07**	**0,0001**
hsa-miR-28-5p	20500158	MIMAT0000085	chr3	+	4,48	0,59	**14,86**	**0,0001**	**0,0053**
hsa-miR-200b-5p	20500555	MIMAT0004571	chr1	+	4,23	0,35	**14,8**	**5,06E-06**	**0,0006**
hsa-miR-4505	20518901	MIMAT0019041	chr14	+	4,08	0,23	**14,39**	**1,69E-07**	**5,56E-05**
hsa-miR-130b-3p	20501181	MIMAT0000691	chr22	+	3,8	0,11	**12,91**	**1,41E-09**	**1,87E-06**
hsa-miR-18a-5p	20500132	MIMAT0000072	chr13	+	3,63	-0,03	**12,64**	**7,34E-06**	**0,0008**
hsa-miR-423-5p	20502123	MIMAT0004748	chr17	+	5,06	1,47	**12,08**	**3,28E-06**	**0,0004**
hsa-miR-378f	20518788	MIMAT0018932	chr1	+	4,16	0,57	**12,06**	**0,0004**	**0,0132**
hsa-miR-7110-5p	20526178	MIMAT0028117	chr3	+	4,83	1,3	**11,55**	**1,62E-08**	**1,07E-05**
hsa-miR-320e	20515627	MIMAT0015072	chr19	-	4,5	1,02	**11,18**	**4,47E-05**	**0,0031**
hsa-miR-4521	20518919	MIMAT0019058	chr17	+	3,46	0,01	**10,94**	**0,0002**	**0,0089**
hsa-miR-409-3p	20502456	MIMAT0001639	chr14	+	4,37	1,01	**10,33**	**5,94E-07**	**0,0001**
hsa-miR-487b-3p	20504218	MIMAT0003180	chr14	+	3,58	0,22	**10,27**	**0,0002**	**0,0089**
hsa-miR-432-5p	20503800	MIMAT0002814	chr14	+	3,38	0,04	**10,12**	**1,25E-05**	**0,0011**
hsa-miR-3613-3p	20517821	MIMAT0017991	chr13	-	3,99	0,65	**10,09**	**0,0007**	**0,0177**
hsa-miR-4492	20518887	MIMAT0019027	chr11	+	6,44	3,14	**9,87**	**7,06E-08**	**3,90E-05**
hsa-miR-1307-3p	20506901	MIMAT0005951	chr10	-	3,6	0,33	**9,65**	**1,82E-05**	**0,0016**
hsa-miR-378i	20518936	MIMAT0019074	chr22	-	4,22	1,02	**9,14**	**0,0005**	**0,0155**
hsa-miR-127-3p	20500771	MIMAT0000446	chr14	+	5,22	2,07	**8,88**	**0,0004**	**0,0132**
hsa-miR-151a-3p	20501287	MIMAT0000757	chr8	-	4,42	1,27	**8,86**	**1,84E-07**	**5,56E-05**
hsa-miR-422a	20502122	MIMAT0001339	chr15	-	3,68	0,53	**8,86**	**7,25E-05**	**0,0042**
hsa-let-7f-5p	20500123	MIMAT0000067	chr9	+	5	1,86	**8,84**	**2,74E-06**	**0,0004**
hsa-miR-23b-5p	20500720	MIMAT0004587	chr9	+	3,3	0,17	**8,71**	**0,0003**	**0,0119**
hsa-miR-1271-5p	20504569	MIMAT0005796	chr5	+	3,5	0,43	**8,38**	**1,06E-05**	**0,001**
hsa-miR-1233-5p	20506781	MIMAT0022943	chr15	-	3,35	0,3	**8,31**	**2,10E-06**	**0,0003**
hsa-miR-4485	20518879	MIMAT0019019	chr11	-	6,44	3,41	**8,21**	**2,46E-09**	**2,72E-06**
hsa-miR-532-5p	20503907	MIMAT0002888	chrX	+	5,26	2,23	**8,18**	**7,98E-07**	**0,0002**
hsa-miR-106b-3p	20501158	MIMAT0004672	chr7	-	3,3	0,31	**7,98**	**4,26E-06**	**0,0005**
hsa-miR-937-5p	20506006	MIMAT0022938	chr8	-	3,61	0,71	**7,43**	**2,26E-05**	**0,0018**
hsa-miR-28-3p	20500159	MIMAT0004502	chr3	+	3,42	0,54	**7,39**	**0,0004**	**0,0125**
hsa-miR-6812-5p	20525585	MIMAT0027524	chr20	+	5	2,15	**7,2**	**1,74E-05**	**0,0015**
hsa-miR-197-3p	20500395	MIMAT0000227	chr1	+	4,04	1,22	**7,07**	**0,0001**	**0,0053**
hsa-miR-4443	20518818	MIMAT0018961	chr3	+	3,15	0,33	**7,06**	**3,56E-05**	**0,0026**
hsa-miR-744-5p	20505787	MIMAT0004945	chr17	+	5,77	2,97	**6,97**	**0,0001**	**0,0053**
hsa-miR-4507	20518904	MIMAT0019044	chr14	-	4,55	1,77	**6,87**	**3,29E-05**	**0,0025**
hsa-miR-3621	20517835	MIMAT0018002	chr9	-	3,77	1,01	**6,81**	**0,0007**	**0,0177**
hsa-miR-181b-5p	20500446	MIMAT0000257	chr1	-	5,42	2,67	**6,76**	**0,0007**	**0,0177**
hsa-miR-1246	20506837	MIMAT0005898	chr2	-	3,27	0,52	**6,7**	**0,0004**	**0,0131**
hsa-miR-1260b	20515591	MIMAT0015041	chr11	+	3,02	0,28	**6,69**	**0,0005**	**0,0151**
hsa-miR-146b-5p	20503793	MIMAT0002809	chr10	+	2,63	-0,1	**6,64**	**5,16E-05**	**0,0034**
hsa-miR-708-5p	20505760	MIMAT0028113	chr19	-	4,94	2,29	**6,24**	**0,0003**	**0,0106**
hsa-miR-425-5p	20502129	MIMAT0003393	chr3	-	5,45	2,83	**6,16**	**4,19E-07**	**0,0001**

hsa-miR-4429	20518801	MIMAT0018944	chr2	-	5,14	2,59	**5,86**	**3,17E-05**	**0,0024**
hsa-miR-3620-5p	20517833	MIMAT0022967	chr1	+	5,03	2,5	**5,8**	**1,10E-06**	**0,0002**
hsa-miR-4750-5p	20519609	MIMAT0019887	chr19	+	3,02	0,49	**5,8**	**0,0002**	**0,008**
hsa-miR-455-3p	20504187	MIMAT0004784	chr9	+	6,78	4,28	**5,67**	**8,46E-07**	**0,0002**
hsa-miR-378c	20517675	MIMAT0016847	chr10	-	5,54	3,05	**5,64**	**4,27E-07**	**0,0001**
hsa-miR-6779-5p	20525519	MIMAT0027458	chr17	+	3,02	0,56	**5,52**	**0,0009**	**0,0203**
hsa-miR-362-5p	20501201	MIMAT0000705	chrX	+	3,09	0,64	**5,45**	**0,0001**	**0,0067**
hsa-miR-6746-5p	20525453	MIMAT0025850	chr19	-	3,19	0,78	**5,33**	**0,0007**	**0,0179**
hsa-miR-4284	20517744	MIMAT0016915	chr7	+	2,78	0,4	**5,2**	**1,14E-05**	**0,0011**
hsa-miR-379-5p	20501244	MIMAT0000733	chr14	+	2,83	0,48	**5,12**	**5,56E-06**	**0,0006**
hsa-miR-21-5p	20500141	MIMAT0000076	chr17	+	2,22	-0,1	**4,98**	**0,0001**	**0,0059**
hsa-miR-34a-5p	20500442	MIMAT0000255	chr1	-	3,27	1,02	**4,76**	**0,0002**	**0,0099**
hsa-miR-151b	20504561	MIMAT0010214	chr14	-	2,7	0,48	**4,68**	**0,0003**	**0,0113**
hsa-miR-24-2-5p	20500149	MIMAT0004497	chr19	-	2,53	0,36	**4,51**	**9,48E-05**	**0,0049**
hsa-miR-6085	20523017	MIMAT0023710	chr15	+	4,13	2	**4,37**	**0,001**	**0,0216**
hsa-miR-342-5p	20501279	MIMAT0004694	chr14	+	2,12	0,01	**4,32**	**0,0001**	**0,0067**
hsa-miR-27b-5p	20500722	MIMAT0004588	chr9	+	2,46	0,35	**4,31**	**0,0007**	**0,018**
hsa-miR-106b-5p	20501157	MIMAT0000680	chr7	-	5,6	3,51	**4,25**	**0,0005**	**0,0155**
hsa-miR-1275	20506872	MIMAT0005929	chr6	-	2,41	0,35	**4,17**	**0,0001**	**0,0057**
hsa-miR-6880-5p	20525721	MIMAT0027660	chr12	-	2,25	0,19	**4,15**	**4,43E-06**	**0,0005**
hsa-let-7g-5p	20500713	MIMAT0000414	chr3	-	5,34	3,32	**4,06**	**0,0001**	**0,0055**
hsa-miR-345-5p	20501312	MIMAT0000772	chr14	+	2,42	0,43	**3,97**	**0,0031**	**0,047**
hsa-miR-134-5p	20500773	MIMAT0000447	chr14	+	2,09	0,1	**3,96**	**2,37E-05**	**0,0019**
hsa-miR-193b-5p	20503807	MIMAT0004767	chr16	+	2,12	0,18	**3,84**	**6,04E-06**	**0,0007**
hsa-miR-4669	20519467	MIMAT0019749	chr9	+	2,27	0,35	**3,78**	**0,0017**	**0,0334**
hsa-miR-20b-5p	20502237	MIMAT0001413	chrX	-	2,64	0,74	**3,73**	**0,0025**	**0,0416**
hsa-miR-6879-5p	20525719	MIMAT0027658	chr11	+	1,94	0,1	**3,57**	**0,0009**	**0,0198**
hsa-miR-125b-2-3p	20500767	MIMAT0004603	chr21	+	2,01	0,19	**3,53**	**0,0004**	**0,0132**
hsa-miR-181a-5p	20500444	MIMAT0000256	chr1	-	6,57	4,8	**3,41**	**0,0005**	**0,0148**
hsa-miR-4728-5p	20519570	MIMAT0019849	chr17	+	2,56	0,81	**3,37**	**0,0028**	**0,0444**
hsa-miR-619-5p	20504364	MIMAT0026622	chr12	-	3,49	1,75	**3,33**	**0,0004**	**0,0137**
hsa-miR-382-5p	20501250	MIMAT0000737	chr14	+	1,89	0,18	**3,28**	**0,0014**	**0,0281**
hsa-miR-15b-5p	20500718	MIMAT0000417	chr3	+	6,27	4,57	**3,23**	**6,29E-05**	**0,0038**
hsa-miR-501-3p	20503878	MIMAT0004774	chrX	+	4,25	2,59	**3,17**	**0,0004**	**0,0138**
hsa-miR-4749-5p	20519607	MIMAT0019885	chr19	+	1,58	-0,09	**3,17**	**0,0031**	**0,047**
hsa-miR-1273g-3p	20520351	MIMAT0022742	chr1	+	9,04	7,39	**3,13**	**4,97E-07**	**0,0001**
hsa-miR-132-3p	20500737	MIMAT0000426	chr17	-	1,65	0,07	**2,99**	**0,0024**	**0,0405**
hsa-miR-15a-5p	20500126	MIMAT0000068	chr13	-	1,55	0,02	**2,91**	**0,0006**	**0,0173**
hsa-miR-2110	20511549	MIMAT0010133	chr10	-	1,92	0,39	**2,9**	**3,45E-05**	**0,0025**
hsa-miR-27b-3p	20500723	MIMAT0000419	chr9	+	7,08	5,57	**2,86**	**9,73E-08**	**4,03E-05**
hsa-miR-23a-5p	20500145	MIMAT0004496	chr19	-	1,49	0	**2,82**	**5,89E-05**	**0,0037**
hsa-miR-4668-5p	20519463	MIMAT0019745	chr9	+	3,92	2,44	**2,79**	**0,0018**	**0,0334**
hsa-let-7i-5p	20500715	MIMAT0000415	chr12	+	6,81	5,36	**2,74**	**3,68E-06**	**0,0004**
hsa-miR-4433-3p	20518807	MIMAT0018949	chr2	+	2,41	0,95	**2,74**	**0,0025**	**0,0416**
hsa-miR-3135b	20518843	MIMAT0018985	chr6	-	1,47	0,04	**2,71**	**0,0005**	**0,015**
hsa-miR-27a-5p	20500156	MIMAT0000084	chr19	-	1,56	0,12	**2,71**	**0,0026**	**0,0426**
hsa-miR-185-5p	20500787	MIMAT0000455	chr22	+	6,54	5,11	**2,69**	**7,70E-05**	**0,0044**
hsa-miR-3911	20518432	MIMAT0018185	chr9	-	2,08	0,67	**2,66**	**0,0024**	**0,0405**
hsa-let-7b-5p	20500115	MIMAT0000063	chr22	+	12,23	10,83	**2,65**	**5,93E-07**	**0,0001**
hsa-miR-500a-5p	20503875	MIMAT0002871	chrX	+	1,63	0,24	**2,63**	**0,0021**	**0,0373**
hsa-miR-423-3p	20502124	MIMAT0001340	chr17	+	6,24	4,87	**2,59**	**0,0004**	**0,0137**
hsa-miR-205-5p	20500462	MIMAT0000266	chr1	+	10,11	8,75	**2,58**	**9,33E-08**	**4,03E-05**
hsa-miR-221-3p	20500484	MIMAT0000278	chrX	-	7,81	6,5	**2,48**	**3,86E-07**	**0,0001**
hsa-miR-4484	20518878	MIMAT0019018	chr10	+	5,26	4,01	**2,39**	**0,0006**	**0,0159**
hsa-miR-1207-5p	20506801	MIMAT0005871	chr8	+	5,18	3,94	**2,37**	**6,52E-05**	**0,0039**
hsa-miR-4286	20517745	MIMAT0016916	chr8	+	1,63	0,39	**2,36**	**0,003**	**0,0459**
hsa-miR-193b-3p	20503808	MIMAT0002819	chr16	+	7,38	6,15	**2,35**	**0,0008**	**0,019**
hsa-miR-93-5p	20500173	MIMAT0000093	chr7	-	7,13	5,94	**2,28**	**8,97E-07**	**0,0002**
hsa-miR-342-3p	20501280	MIMAT0000753	chr14	+	7,46	6,3	**2,24**	**0,0002**	**0,0095**

hsa-miR-378a-3p	20501243	MIMAT0000732	chr5	+	7,29	6,13	**2,23**	**2,99E-06**	**0,0004**
hsa-miR-182-5p	20500450	MIMAT0000259	chr7	-	5,81	4,67	**2,21**	**0,0002**	**0,0069**
hsa-miR-23a-3p	20500146	MIMAT0000078	chr19	-	10,59	9,45	**2,19**	**7,68E-08**	**3,92E-05**
hsa-miR-16-5p	20500128	MIMAT0000069	chr13	-	7,93	6,8	**2,19**	**0,0001**	**0,0059**
hsa-miR-92a-3p	20500171	MIMAT0000092	chr13	+	8,96	7,87	**2,13**	**7,29E-05**	**0,0042**
hsa-miR-17-5p	20500130	MIMAT0000070	chr13	+	7,2	6,12	**2,12**	**8,10E-06**	**0,0008**
hsa-miR-6819-5p	20525599	MIMAT0027538	chr22	-	1,48	0,4	**2,11**	**0,0004**	**0,0138**
hsa-miR-106a-5p	20500194	MIMAT0000103	chrX	-	6,91	5,85	**2,09**	**1,59E-06**	**0,0003**
hsa-miR-22-3p	20500144	MIMAT0000077	chr17	-	6,55	5,49	**2,08**	**4,65E-05**	**0,0031**
hsa-miR-4739	20519589	MIMAT0019868	chr17	-	5,92	4,87	**2,07**	**0,0001**	**0,0067**
hsa-miR-203a	20500459	MIMAT0000264	chr14	+	9,02	7,98	**2,06**	**2,40E-06**	**0,0004**
hsa-miR-17-3p	20500131	MIMAT0000071	chr13	+	0,91	-0,1	**2,01**	**0,0003**	**0,0119**
hsa-miR-99b-5p	20501176	MIMAT0000689	chr19	+	6,76	5,76	**2,01**	**0,0005**	**0,0145**
hsa-miR-4530	20518931	MIMAT0019069	chr19	-	8,27	9,38	**-2,15**	**1,77E-07**	**5,56E-05**
hsa-miR-6743-5p	20525448	MI0022554	chr19	-	7,05	8,19	**-2,21**	**1,50E-06**	**0,0003**
hsa-miR-8075	20529785	MIMAT0031002	chr13	+	4,89	6,23	**-2,53**	**7,37E-06**	**0,0008**

Table S2

Transcript ID	array ID	Accession	Chromosome	Strand	PV (log2)	controls (log2)	PV vs controls		
							**Fold Change**	**P-value**	**FDR adj p-value**
hsa-miR-31-5p	20500164	MIMAT0000089	chr9	-	7,23	0,11	**139,67**	**5,40E-21**	**3,58E-17**
hsa-miR-146a-5p	20500778	MIMAT0000449	chr5	+	6,44	0,73	**52,31**	**9,49E-10**	**7,87E-07**
hsa-miR-7977	20529568	MIMAT0031180	chr3	+	5,99	0,64	**40,81**	**4,04E-07**	**9,93E-05**
hsa-miR-3651	20517902	MIMAT0018071	chr9	-	5,57	0,53	**32,95**	**3,46E-08**	**1,64E-05**
hsa-miR-2115-5p	20511558	MIMAT0011158	chr3	-	4,91	0,3	**24,46**	**6,57E-13**	**2,18E-09**
hsa-miR-378f	20518788	MIMAT0018932	chr1	+	5,13	0,57	**23,6**	**0,0002**	**0,0117**
hsa-miR-1233-5p	20506781	MIMAT0022943	chr15	-	4,82	0,3	**22,96**	**2,34E-10**	**2,47E-07**
hsa-miR-6126	20524036	MIMAT0024599	chr16	-	4,58	0,07	**22,7**	**0,0002**	**0,0125**
hsa-miR-5100	20521785	MIMAT0022259	chr10	+	6,39	2,05	**20,2**	**9,35E-09**	**6,07E-06**
hsa-miR-3175	20515603	MIMAT0015052	chr15	+	4,67	0,36	**19,88**	**1,14E-06**	**0,0002**
hsa-miR-25-3p	20500151	MIMAT0000081	chr7	-	5,11	1,1	**16,15**	**9,70E-06**	**0,0014**
hsa-miR-7110-5p	20526178	MIMAT0028117	chr3	+	5,19	1,3	**14,87**	**1,52E-08**	**8,41E-06**
hsa-miR-200b-3p	20500556	MIMAT0000318	chr1	+	4,56	0,74	**14,09**	**1,40E-06**	**0,0003**
hsa-miR-200b-5p	20500555	MIMAT0004571	chr1	+	3,91	0,35	**11,85**	**0,0003**	**0,0139**
hsa-miR-28-5p	20500158	MIMAT0000085	chr3	+	4,15	0,59	**11,78**	**9,74E-05**	**0,0076**
hsa-miR-4443	20518818	MIMAT0018961	chr3	+	3,88	0,33	**11,74**	**2,67E-05**	**0,0027**
hsa-miR-423-5p	20502123	MIMAT0004748	chr17	+	4,98	1,47	**11,39**	**2,72E-05**	**0,0027**
hsa-miR-5189-3p	20520569	MIMAT0027088	chr16	+	3,64	0,14	**11,3**	**1,57E-05**	**0,002**
hsa-miR-6812-5p	20525585	MIMAT0027524	chr20	+	5,59	2,15	**10,86**	**1,21E-07**	**4,00E-05**
hsa-let-7f-5p	20500123	MIMAT0000067	chr9	+	5,29	1,86	**10,78**	**3,89E-07**	**9,91E-05**
hsa-miR-1260b	20515591	MIMAT0015041	chr11	+	3,65	0,28	**10,34**	**0,0001**	**0,0088**
hsa-miR-4505	20518901	MIMAT0019041	chr14	+	3,57	0,23	**10,11**	**5,86E-06**	**0,0009**
hsa-miR-320e	20515627	MIMAT0015072	chr19	-	4,32	1,02	**9,8**	**0,0003**	**0,014**
hsa-miR-4521	20518919	MIMAT0019058	chr17	+	3,28	0,01	**9,64**	**0,0002**	**0,0123**
hsa-miR-6746-5p	20525453	MIMAT0025850	chr19	-	4,04	0,78	**9,6**	**1,32E-05**	**0,0017**
hsa-miR-937-5p	20506006	MIMAT0022938	chr8	-	3,95	0,71	**9,44**	**6,80E-05**	**0,0057**
hsa-miR-378c	20517675	MIMAT0016847	chr10	-	6,27	3,05	**9,32**	**9,41E-09**	**6,07E-06**
hsa-miR-18a-5p	20500132	MIMAT0000072	chr13	+	3,19	-0,03	**9,32**	**3,72E-06**	**0,0006**
hsa-miR-4728-5p	20519570	MIMAT0019849	chr17	+	3,9	0,81	**8,54**	**1,74E-07**	**5,48E-05**
hsa-miR-4507	20518904	MIMAT0019044	chr14	-	4,81	1,77	**8,22**	**0,0004**	**0,0176**
hsa-miR-532-3p	20503908	MIMAT0004780	chrX	+	3,43	0,55	**7,37**	**0,0003**	**0,0152**
hsa-miR-378i	20518936	MIMAT0019074	chr22	-	3,91	1,02	**7,37**	**0,0003**	**0,016**
hsa-miR-4284	20517744	MIMAT0016915	chr7	+	3,28	0,4	**7,35**	**1,18E-06**	**0,0002**
hsa-miR-151a-3p	20501287	MIMAT0000757	chr8	-	4,13	1,27	**7,25**	**4,73E-06**	**0,0008**
hsa-miR-6880-5p	20525721	MIMAT0027660	chr12	-	3,05	0,19	**7,21**	**6,86E-07**	**0,0001**
hsa-miR-3620-5p	20517833	MIMAT0022967	chr1	+	5,32	2,5	**7,08**	**6,34E-07**	**0,0001**
hsa-miR-4429	20518801	MIMAT0018944	chr2	-	5,39	2,59	**6,98**	**8,21E-06**	**0,0013**
hsa-miR-708-5p	20505760	MIMAT0028113	chr19	-	5,09	2,29	**6,94**	**0,0002**	**0,0126**
hsa-miR-1246	20506837	MIMAT0005898	chr2	-	3,27	0,52	**6,7**	**0,0013**	**0,0424**
hsa-miR-4492	20518887	MIMAT0019027	chr11	+	5,88	3,14	**6,69**	**4,74E-07**	**0,0001**
hsa-miR-422a	20502122	MIMAT0001339	chr15	-	3,26	0,53	**6,64**	**0,0002**	**0,0121**
hsa-miR-4669	20519467	MIMAT0019749	chr9	+	3,05	0,35	**6,5**	**0,0001**	**0,0098**
hsa-miR-4485	20518879	MIMAT0019019	chr11	-	6,03	3,41	**6,17**	**3,20E-08**	**1,63E-05**
hsa-miR-744-5p	20505787	MIMAT0004945	chr17	+	5,59	2,97	**6,13**	**0,0002**	**0,0108**
hsa-miR-532-5p	20503907	MIMAT0002888	chrX	+	4,77	2,23	**5,83**	**0,0002**	**0,0122**
hsa-miR-425-5p	20502129	MIMAT0003393	chr3	-	5,35	2,83	**5,75**	**4,20E-07**	**9,94E-05**
hsa-miR-34a-5p	20500442	MIMAT0000255	chr1	-	3,47	1,02	**5,45**	**0,0003**	**0,0152**
hsa-miR-6879-5p	20525719	MIMAT0027658	chr11	+	2,47	0,1	**5,14**	**0,0001**	**0,0102**
hsa-miR-1273g-3p	20520351	MIMAT0022742	chr1	+	9,72	7,39	**5,02**	**1,01E-08**	**6,07E-06**
hsa-miR-1307-3p	20506901	MIMAT0005951	chr10	-	2,64	0,33	**4,97**	**0,001**	**0,0349**
hsa-miR-130b-3p	20501181	MIMAT0000691	chr22	+	2,36	0,11	**4,75**	**1,65E-05**	**0,002**
hsa-miR-27b-5p	20500722	MIMAT0004588	chr9	+	2,55	0,35	**4,6**	**0,0009**	**0,0337**
hsa-miR-6831-5p	20525623	MIMAT0027562	chr5	-	2,93	0,8	**4,37**	**1,55E-05**	**0,002**
hsa-let-7g-5p	20500713	MIMAT0000414	chr3	-	5,31	3,32	**3,97**	**0,0006**	**0,0255**

hsa-miR-106b-5p	20501157	MIMAT0000680	chr7	-	5,49	3,51	**3,94**	**0,0015**	**0,0467**
hsa-miR-409-3p	20502456	MIMAT0001639	chr14	+	2,97	1,01	**3,9**	**0,0003**	**0,0137**
hsa-miR-3911	20518432	MIMAT0018185	chr9	-	2,6	0,67	**3,81**	**0,0002**	**0,0118**
hsa-miR-362-5p	20501201	MIMAT0000705	chrX	+	2,56	0,64	**3,79**	**0,0004**	**0,0176**
hsa-miR-1275	20506872	MIMAT0005929	chr6	-	2,24	0,35	**3,7**	**0,0005**	**0,0212**
hsa-miR-27b-3p	20500723	MIMAT0000419	chr9	+	7,42	5,57	**3,6**	**4,93E-08**	**1,89E-05**
hsa-miR-4286	20517745	MIMAT0016916	chr8	+	2,15	0,39	**3,39**	**0,0002**	**0,0107**
hsa-miR-146b-5p	20503793	MIMAT0002809	chr10	+	1,66	-0,1	**3,38**	**0,0009**	**0,0337**
hsa-miR-455-3p	20504187	MIMAT0004784	chr9	+	6,01	4,28	**3,33**	**4,41E-05**	**0,004**
hsa-miR-6085	20523017	MIMAT0023710	chr15	+	3,71	2	**3,28**	**0,0009**	**0,0338**
hsa-miR-15a-5p	20500126	MIMAT0000068	chr13	-	1,71	0,02	**3,24**	**0,0003**	**0,0161**
hsa-miR-185-5p	20500787	MIMAT0000455	chr22	+	6,77	5,11	**3,16**	**0,0002**	**0,0124**
hsa-miR-1207-5p	20506801	MIMAT0005871	chr8	+	5,4	3,94	**2,75**	**1,61E-05**	**0,002**
hsa-miR-93-5p	20500173	MIMAT0000093	chr7	-	7,33	5,94	**2,63**	**4,61E-07**	**0,0001**
hsa-miR-6808-3p	20525578	MIMAT0027517	chr1	-	1,73	0,34	**2,62**	**5,69E-05**	**0,005**
hsa-miR-205-5p	20500462	MIMAT0000266	chr1	+	10,1	8,75	**2,56**	**2,20E-07**	**6,62E-05**
hsa-miR-378a-3p	20501243	MIMAT0000732	chr5	+	7,43	6,13	**2,45**	**6,80E-07**	**0,0001**
hsa-miR-203a	20500459	MIMAT0000264	chr14	+	9,26	7,98	**2,43**	**4,90E-08**	**1,89E-05**
hsa-miR-194-5p	20500797	MIMAT0000460	chr1	-	1,6	0,31	**2,43**	**0,0012**	**0,041**
hsa-miR-106a-5p	20500194	MIMAT0000103	chrX	-	7,11	5,85	**2,41**	**1,15E-07**	**4,00E-05**
hsa-let-7i-5p	20500715	MIMAT0000415	chr12	+	6,63	5,36	**2,41**	**2,68E-05**	**0,0027**
hsa-miR-17-5p	20500130	MIMAT0000070	chr13	+	7,32	6,12	**2,3**	**5,13E-08**	**1,89E-05**
hsa-miR-182-5p	20500450	MIMAT0000259	chr7	-	5,85	4,67	**2,27**	**4,05E-05**	**0,0037**
hsa-miR-629-5p	20504379	MIMAT0004810	chr15	-	1,11	-0,07	**2,26**	**0,0015**	**0,0462**
hsa-miR-22-3p	20500144	MIMAT0000077	chr17	-	6,62	5,49	**2,18**	**0,0002**	**0,0132**
hsa-miR-342-3p	20501280	MIMAT0000753	chr14	+	7,4	6,3	**2,15**	**0,001**	**0,037**
hsa-miR-4739	20519589	MIMAT0019868	chr17	-	5,94	4,87	**2,11**	**0,0008**	**0,0301**

Table S3

Transcript ID	array ID	Accession	Chromosome	Strand	AD (log2)	PV (log2)	AD vs PV		
							Fold Change	P-value	FDR adj p-value
hsa-miR-487b-3p	20504218	MIMAT0003180	chr14	+	3,58	1,1	5,57	0,0195	0,6932
hsa-miR-432-5p	20503800	MIMAT0002814	chr14	+	3,38	1,1	4,87	0,0013	0,4417
hsa-miR-92b-3p	20504274	MIMAT0003218	chr1	+	4,72	2,6	4,34	0,0126	0,6799
hsa-miR-1271-5p	20504569	MIMAT0005796	chr5	+	3,5	1,48	4,06	0,0083	0,6799
hsa-miR-106b-3p	20501158	MIMAT0004672	chr7	-	3,3	1,63	3,19	0,0073	0,6559
hsa-miR-342-5p	20501279	MIMAT0004694	chr14	+	2,12	0,58	2,9	0,0348	0,7177
hsa-miR-193b-5p	20503807	MIMAT0004767	chr16	+	2,12	0,63	2,8	0,0003	0,4365
hsa-miR-24-2-5p	20500149	MIMAT0004497	chr19	-	2,53	1,05	2,79	0,0165	0,6799
hsa-miR-6802-5p	20525565	MIMAT0027504	chr19	-	1,91	0,44	2,77	0,0295	0,7161
hsa-miR-130b-3p	20501181	MIMAT0000691	chr22	+	3,8	2,36	2,72	0,0009	0,4417
hsa-miR-409-3p	20502456	MIMAT0001639	chr14	+	4,37	2,97	2,65	0,0301	0,7161
hsa-miR-379-5p	20501244	MIMAT0000733	chr14	+	2,83	1,63	2,3	0,0038	0,6048
hsa-miR-134-5p	20500773	MIMAT0000447	chr14	+	2,09	0,91	2,26	0,0067	0,6298
hsa-miR-149-5p	20500780	MIMAT0004609	chr2	+	5,49	4,4	2,14	0,0221	0,7085
hsa-miR-31-5p	20500164	MIMAT0000089	chr9	-	6,2	7,23	-2,05	0,0007	0,4417
hsa-miR-6831-5p	20525623	MIMAT0027562	chr5	-	1,77	2,93	-2,24	0,0064	0,6298
hsa-miR-146a-5p	20500778	MIMAT0000449	chr5	+	5,18	6,44	-2,39	0,0388	0,7253
hsa-miR-4728-5p	20519570	MIMAT0019849	chr17	+	2,56	3,9	-2,53	0,0009	0,4417
hsa-miR-1233-5p	20506781	MIMAT0022943	chr15	-	3,35	4,82	-2,76	0,0009	0,4417

Table S4

arms	gene_name	chr	start_hg38	stop_hg38	rsID	chromosome.1	pos_hg38	reference_allele	other_allele	eaf	European_N	beta	se	p,value < 0.05/10^6	AllEthnicities_N	minuslog10pvalue
hsa-let-7f-5p,hsa-let-7g-5p,hsa-let-7i-5p	IL13	5	132656522	132661110	rs12188917	chr5	132655393	C	T	0.205033	40530	0,170	0,022	2,89E-15	54323	14,539
hsa-let-7f-5p,hsa-let-7g-5p,hsa-let-7i-5p	IL13	5	132656522	132661110	rs2040703	chr5	132636566	G	C	0.218375	40529	0,153	0,020	2,61E-14	54326	13,583
hsa-let-7f-5p,hsa-let-7g-5p,hsa-let-7i-5p	IL13	5	132656522	132661110	rs2074369	chr5	132637971	C	T	0.218053	40529	0,153	0,020	3,10E-14	54323	13,509
hsa-let-7f-5p,hsa-let-7g-5p,hsa-let-7i-5p	IL13	5	132656522	132661110	rs2040704	chr5	132637485	G	A	0.218323	40530	0,152	0,020	3,88E-14	54327	13,411
hsa-let-7f-5p,hsa-let-7g-5p,hsa-let-7i-5p	IL13	5	132656522	132661110	rs6866095	chr5	132633253	C	T	0.214158	40530	0,152	0,020	6,59E-14	54327	13,181
hsa-let-7f-5p,hsa-let-7g-5p,hsa-let-7i-5p	IL13	5	132656522	132661110	rs6596087	chr5	132632917	A	G	0.215121	40531	0,151	0,020	9,57E-14	54328	13,019
hsa-let-7f-5p,hsa-let-7g-5p,hsa-let-7i-5p	IL13	5	132656522	132661110	rs6596090	chr5	132652723	A	G	0.216627	40529	0,154	0,021	9,68E-14	53060	13,014
hsa-let-7f-5p,hsa-let-7g-5p,hsa-let-7i-5p	IL13	5	132656522	132661110	rs7737470	chr5	132638371	A	T	0.213619	40530	0,151	0,020	1,16E-13	54327	12,936
hsa-let-7f-5p,hsa-let-7g-5p,hsa-let-7i-5p	IL13	5	132656522	132661110	rs6872131	chr5	132637690	G	A	0.214706	40529	0,150	0,020	1,20E-13	54326	12,921
hsa-let-7f-5p,hsa-let-7g-5p,hsa-let-7i-5p	IL13	5	132656522	132661110	rs1800925	chr5	132657117	T	C	0.199556	40529	0,160	0,022	1,37E-13	54320	12,863
hsa-let-7f-5p,hsa-let-7g-5p,hsa-let-7i-5p	IL13	5	132656522	132661110	rs6871536	chr5	132634182	C	T	0.214458	40834	0,148	0,020	1,62E-13	54629	12,790
hsa-let-7f-5p,hsa-let-7g-5p,hsa-let-7i-5p	IL13	5	132656522	132661110	rs3091307	chr5	132653444	G	A	0.216765	40529	0,153	0,021	1,72E-13	54326	12,764
hsa-let-7f-5p,hsa-let-7g-5p,hsa-let-7i-5p	IL13	5	132656522	132661110	rs12653750	chr5	132636210	T	C	0.212717	40835	0,148	0,020	2,23E-13	54627	12,652
hsa-let-7f-5p,hsa-let-7g-5p,hsa-let-7i-5p	IL13	5	132656522	132661110	5:131974177:INDEL	chr5	132638485	D	R	0.212408	40834	0,148	0,020	2,72E-13	45193	12,565
hsa-let-7f-5p,hsa-let-7g-5p,hsa-let-7i-5p	IL13	5	132656522	132661110	5:131980467:INDEL	chr5	132644775	I	R	0.222147	40529	0,149	0,020	3,02E-13	44888	12,520
hsa-let-7f-5p,hsa-let-7g-5p,hsa-let-7i-5p	IL13	5	132656522	132661110	rs62385262	chr5	132646679	T	C	0.21199	40530	0,149	0,021	3,67E-13	54327	12,435
hsa-let-7f-5p,hsa-let-7g-5p,hsa-let-7i-5p	IL13	5	132656522	132661110	rs60632435	chr5	132646823	A	T	0.213974	40529	0,149	0,021	4,19E-13	54325	12,378
hsa-let-7f-5p,hsa-let-7g-5p,hsa-let-7i-5p	IL13	5	132656522	132661110	rs62385261	chr5	132646431	A	T	0.214675	40530	0,148	0,020	4,93E-13	54326	12,307
hsa-let-7f-5p,hsa-let-7g-5p,hsa-let-7i-5p	IL13	5	132656522	132661110	rs2240032	chr5	132641435	T	C	0.211889	40834	0,146	0,020	5,01E-13	54626	12,300
hsa-let-7f-5p,hsa-let-7g-5p,hsa-let-7i-5p	IL13	5	132656522	132661110	5:131969278:INDEL	chr5	132633586	D	R	0.182324	40530	0,163	0,023	5,72E-13	44889	12,243
hsa-let-7f-5p,hsa-let-7g-5p,hsa-let-7i-5p	IL13	5	132656522	132661110	rs1881457	chr5	132656717	C	A	0.204358	40530	0,155	0,022	9,65E-13	54326	12,015
hsa-let-7f-5p,hsa-let-7g-5p,hsa-let-7i-5p	IL13	5	132656522	132661110	rs3091308	chr5	132655340	A	G	0.204097	40529	0,154	0,022	1,16E-12	54316	11,936
hsa-let-7f-5p,hsa-let-7g-5p,hsa-let-7i-5p	IL13	5	132656522	132661110	rs67006560	chr5	132655964	T	C	0.198898	40530	0,151	0,022	3,16E-12	54316	11,500
hsa-let-7f-5p,hsa-let-7g-5p,hsa-let-7i-5p	IL13	5	132656522	132661110	rs7700346	chr5	132647734	A	G	0.204667	40529	0,146	0,021	4,71E-12	54309	11,327
hsa-let-7f-5p,hsa-let-7g-5p,hsa-let-7i-5p	IL13	5	132656522	132661110	rs115008099	chr5	132656189	T	C	0.200425	40530	0,149	0,022	6,67E-12	54327	11,176
hsa-let-7f-5p,hsa-let-7g-5p,hsa-let-7i-5p	IL13	5	132656522	132661110	rs2158177	chr5	132648366	G	A	0.203568	40530	0,143	0,021	1,35E-11	54327	10,870
hsa-let-7f-5p,hsa-let-7g-5p,hsa-let-7i-5p	IL13	5	132656522	132661110	rs72797378	chr5	132651233	T	A	0.202582	40530	0,144	0,021	1,60E-11	54327	10,796
hsa-let-7f-5p,hsa-let-7g-5p,hsa-let-7i-5p	IL13	5	132656522	132661110	rs10056700	chr5	132631790	C	T	0.27113	40529	0,128	0,019	2,22E-11	54326	10,654
hsa-let-7f-5p,hsa-let-7g-5p,hsa-let-7i-5p	IL13	5	132656522	132661110	rs10066662	chr5	132685304	A	G	0.722908	39063	-0,131	0,020	4,17E-11	52844	10,380
hsa-let-7f-5p,hsa-let-7g-5p,hsa-let-7i-5p	IL13	5	132656522	132661110	rs2243293	chr5	132684107	A	G	0.722799	39063	-0,130	0,020	4,64E-11	52860	10,333
hsa-let-7f-5p,hsa-let-7g-5p,hsa-let-7i-5p	IL13	5	132656522	132661110	rs35081016	chr5	132684610	G	A	0.722875	39063	-0,130	0,020	5,22E-11	52860	10,282
hsa-let-7f-5p,hsa-let-7g-5p,hsa-let-7i-5p	IL13	5	132656522	132661110	rs20541	chr5	132660272	G	A	0.791106	40835	-0,132	0,020	8,78E-11	54632	10,057
hsa-let-7f-5p,hsa-let-7g-5p,hsa-let-7i-5p	IL13	5	132656522	132661110	rs1295686	chr5	132660151	C	T	0.786013	40835	-0,130	0,020	9,28E-11	54627	10,032
hsa-let-7f-5p,hsa-let-7g-5p,hsa-let-7i-5p	IL13	5	132656522	132661110	rs6885996	chr5	132685604	A	C	0.714025	39063	-0,127	0,020	1,09E-10	52860	9,963
hsa-let-7f-5p,hsa-let-7g-5p,hsa-let-7i-5p	IL13	5	132656522	132661110	rs10066660	chr5	132685298	A	G	0.714036	39063	-0,127	0,020	1,12E-10	52860	9,951
hsa-let-7f-5p,hsa-let-7g-5p,hsa-let-7i-5p	IL13	5	132656522	132661110	rs2227284	chr5	132677033	G	T	0.71512	39063	-0,125	0,020	1,61E-10	52857	9,793
hsa-let-7f-5p,hsa-let-7g-5p,hsa-let-7i-5p	IL13	5	132656522	132661110	rs2227282	chr5	132677487	G	C	0.714919	39064	-0,124	0,019	2,30E-10	52858	9,638
hsa-let-7f-5p,hsa-let-7g-5p,hsa-let-7i-5p	IL13	5	132656522	132661110	rs2243294	chr5	132684123	A	G	0.703247	39063	-0,122	0,020	6,11E-10	52860	9,214
hsa-let-7f-5p,hsa-let-7g-5p,hsa-let-7i-5p	IL13	5	132656522	132661110	rs848	chr5	132660808	C	A	0.794193	40530	-0,125	0,021	1,55E-09	54320	8,810
hsa-let-7f-5p,hsa-let-7g-5p,hsa-let-7i-5p	IL13	5	132656522	132661110	rs847	chr5	132660977	C	T	0.790878	39064	-0,126	0,021	1,63E-09	52856	8,788
hsa-let-7f-5p,hsa-let-7g-5p,hsa-let-7i-5p	IL13	5	132656522	132661110	rs2243204	chr5	132663802	T	C	0.085139	40530	0,182	0,030	1,72E-09	54323	8,764
hsa-let-7f-5p,hsa-let-7g-5p,hsa-let-7i-5p	IL13	5	132656522	132661110	rs2243208	chr5	132665459	G	A	0.084673	40530	0,177	0,031	1,03E-08	54325	7,987
hsa-miR-28-3p,hsa-miR-28-5p	STAT5B	17	42199177	42288437	rs8066625	chr17	42238611	A	G	0.107212	40529	0,176	0,032	3,84E-08	54316	7,416
hsa-miR-130b-3p	IRF1	5	132481609	132490789	rs2706392	chr5	132508365	T	C	0.188768	40529	0,121	0,022	3,97E-08	54325	7,401
hsa-let-7f-5p,hsa-let-7g-5p,hsa-let-7i-5p	IL13	5	132656522	132661110	rs62385260	chr5	132631848	A	G	0.209919	34565	0,122	0,022	4,63E-08	48362	7,334

Table S5

Transcript ID	array ID	Accession	Chromosome	Strand	AD (log2)	controls (log2)	AD vs controls		
							Fold Change	P-value	FDR adj P-value
hsa-miR-3613-3p	20517821	MIMAT0017991	chr13	-	3,87	2,63	2,36	0,0005	0,3577
hsa-miR-4668-5p	20519463	MIMAT0019745	chr9	+	2,78	1,62	2,24	0,0057	0,7472
hsa-miR-3921	20518446	MIMAT0018196	chr3	-	2,15	1,12	2,04	0,0017	0,6137
hsa-miR-3201	20515646	MIMAT0015086	chr22	+	1,31	0,47	1,78	0,006	0,7472
hsa-let-7a-5p	20500112	MIMAT0000062	chr11	-	0,11	0,75	-1,56	0,0003	0,3577
hsa-miR-6511b-5p	20525386	MIMAT0025847	chr16	-	1,71	2,5	-1,74	0,0185	0,8255
hsa-miR-122-5p	20500726	MIMAT0000421	chr18	+	0,00	0,93	-1,9	0,01497	0,8913
hsa-let-7c-5p	20500117	MIMAT0000064	chr21	+	0,11	1,18	-2,1	0,0003	0,3577
hsa-miR-4487	20518881	MIMAT0019021	chr11	+	1,83	3,6	-3,42	0,0029	0,6687

Table S6

Transcript ID	array ID	Accession	Chromosome	Strand	PV (log2)	controls (log2)	PV vs controls		
							Fold Change	P-value	FDR adj P-value
hsa-let-7a-5p	20500112	MIMAT0000062	chr11	-	0,21	0,75	-1,46	0,0057	0,9995
hsa-let-7c-5p	20500117	MIMAT0000064	chr21	+	0,24	1,18	-1,92	0,0005	0,9995

Table S7

Transcript ID	array ID	Accession	Chromosome	Strand	AD (log2)	PV (log2)	AD vs PV		
							Fold Change	P-value	FDR adj P-value
hsa-miR-3613-3p	20517821	MIMAT0017991	chr13	-	3,87	2,25	3,07	0,0001	0,3626
hsa-miR-297	20506022	MIMAT0004450	chr4	-	1,87	0,35	2,86	0,0475	0,7601
hsa-miR-4668-5p	20519463	MIMAT0019745	chr9	+	2,78	1,65	2,19	0,0112	0,662
hsa-miR-3201	20515646	MIMAT0015086	chr22	+	1,31	0,38	1,9	0,0031	0,5748
hsa-miR-3921	20518446	MIMAT0018196	chr3	-	2,15	1,34	1,77	0,0047	0,6003
hsa-miR-3148	20515562	MIMAT0015021	chr8	-	0,89	0,39	1,42	0,0141	0,6992
hsa-miR-122-5p	20500726	MIMAT0000421	chr18	+	0	0,51	-1,42	0,0449	0,7581
hsa-miR-4487	20518881	MIMAT0019021	chr11	+	1,83	3,26	-2,69	0,0166	0,7127
